# Brain Cancer: Molecular Alterations and Emerging Trends in Neuropharmacology

**DOI:** 10.3390/ijms27114880

**Published:** 2026-05-28

**Authors:** Beata Leskova, Ilaria D’Agostino, Simona Mattova, Nicol Urbanska, Alzbeta Blicharova, Patrik Simko, Aylin Toplu, Muhammet Karaman, Terezia Kiskova-Simkova

**Affiliations:** 1 Department of Pharmacology, Faculty of Medicine, Pavol Jozef Safarik University in Kosice, 040 01 Kosice, Slovakia; beata.leskova@student.upjs.sk; 2 Department of Pharmacy, University of Pisa, 56126 Pisa, Italy; ilaria.dagostino@unipi.it; 3 Department of Pathology, Faculty of Medicine, Pavol Jozef Safarik University in Kosice, 040 01 Kosice, Slovakianicol.urbanska@student.upjs.sk (N.U.); alzbeta.blicharova@upjs.sk (A.B.); patrik.simko@student.upjs.sk (P.S.); 4 Department of Medical Pharmacology, School of Medicine, Health Sciences University, 34668 Istanbul, Türkiye; aylin.toplu@sbu.edu.tr; 5 Department of Biology, Faculty of Science, Dokuz Eylul University, 35390 Izmir, Türkiye; muhammet.karaman@deu.edu.tr

**Keywords:** brain cancer, genetic mutations, epigenetics, multi-omics, artificial intelligence

## Abstract

Central nervous system (CNS) tumors represent a heterogeneous group of neoplasms associated with significant morbidity and mortality despite their relatively low incidence. Advances in the fifth edition of the World Health Organization (WHO) classification have emphasized the integration of histopathological, immunohistochemical, and molecular features, fundamentally transforming diagnostic and prognostic frameworks in neuro-oncology. This manuscript aims to provide an overview of CNS tumor biology, focusing on key diagnostic markers, genetic and epigenetic alterations, and emerging therapeutic strategies. It further describes recent advances in multi-omics approaches and artificial intelligence, which enable deeper characterization of tumor heterogeneity and support the development of precision medicine strategies. Finally, current and emerging therapeutic modalities, including combination therapies, targeted treatments, and novel molecular targets, are examined with emphasis on overcoming resistance mechanisms and improving clinical outcomes. Overall, the integration of molecular biology, advanced diagnostics, and innovative therapeutic approaches represents a critical step toward personalized management of CNS tumors and improved patient survival.

## 1. Introduction

Central nervous system (CNS) tumors originate in different cell types and account for approximately 2% of all cancers. CNS tumors, particularly glioblastoma (GBM), IDH-wildtype, a CNS WHO grade 4 adult-type diffuse glioma, which represents the most common malignant primary brain and other CNS tumor in adults, represent a profound clinical challenge with a high mortality rate and a survival of 2.6%.While traditional epidemiological data heavily rely on older cohorts, large-scale genomic studies, including The Cancer Genome Atlas (TCGA)-derived analyses, have confirmed that survival remains poor despite multimodal treatment regimens.

The age-adjusted incidence rate was 3.5 per 100,000 people, with the highest rates observed in Southern and Western Europe [1]. Of these, primary brain tumors account for approximately 95% of CNS tumors [2]. These tumors represent a heterogeneous group and are stratified into distinct malignancy grades according to the World Health Organization (WHO) classification criteria. There are over 100 types of primary CNS tumors listed by the WHO International Classification of Diseases Oncology, and no dominant risk factor has been identified [3]. Malignant brain tumor incidence is highest in populations of predominantly European ancestry and in individuals with higher socioeconomic status [4].

CNS tumors are the most common cancer in children aged 0–14 years old and the second most common in 15–19 years old [5]. The incidence is highest for 5-year-olds and younger. The majority represent malignant tumors, especially gliomas, embryonal tumors, and germ cell tumors; the most common tumors of the pituitary gland are benign. Despite the high incidence and clinical significance of these tumors, their underlying etiology remains poorly understood [6]. To date, only two major risk factors have been consistently recognized: single-gene inherited disorders, for which available evidence remains limited due to the small number of studies conducted, and exposure to ionizing radiation, which demonstrates a well-established dose–response relationship [5]. In addition, several emerging factors warrant further investigation, including birth weight, as current evidence suggests that higher birth weight may be associated with an increased risk of CNS tumors, as well as non-chromosomal structural birth defects affecting the nervous system [5].

In adults, CNS tumors are the 8th most common cancer [7]. The majority of them are non-malignant, mainly meningioma and tumors of the pituitary gland; among malignant tumors, the most common are gliomas [5]. Despite extensive investigation into potential environmental contributors, high-dose ionizing radiation remains the only consistently established environmental risk factor. Many other risk factors are under investigation, for example, the use of mobile phones with their radiofrequency field, and low-frequency magnetic fields or power lines. However, no significant association emerged between the mentioned factors and risk of any type of brain tumor [8]. Instead, only limited data are available about infectious agents, namely the polyomavirus family [9] and protozoan *Toxoplasma gondii* [10]. On the contrary, an inverse association between previous varicella-zoster virus infection and glioma risk has been reported, potentially reflecting immune-mediated mechanisms. [11]. The same pathomechanism is hypothesized in allergies, reducing glioma risk. Some of the patients with brain cancer have a family history of brain tumors. Several hereditary cancer syndromes are associated with an increased risk of brain tumors, including neurofibromatosis types 1 and 2, tuberous sclerosis complex, and Li-Fraumeni syndrome. Typically, mutated genes in brain tumors are isocitrate dehydrogenase 1 and 2 (IDH1/2), telomerase reverse transcriptase (TERT), epidermal growth factor receptor (EGFR), and cyclin-dependent kinase inhibitor 2A (CDKN2A). All these mutated genes are found mainly in various types of gliomas [5].

This review argues that the current management of CNS tumors is moving from a morphology-centered framework toward a dynamic, multi-layered model in which histology, molecular profiling, spatial heterogeneity, therapeutic vulnerabilities, and computational tools are interpreted together. However, diagnostic markers are not always robust, molecular classifications are rapidly evolving, and many innovative therapeutic strategies remain highly limited by blood-brain barrier (BBB) penetration, intratumoral heterogeneity, and incomplete clinical validation.

## 2. Classification and Immunohistochemical Features of CNS Tumors

### 2.1. WHO 2021 Classification

Diagnosis and classification of CNS tumors are based on the 5 edition of the Central Nervous System Tumors volume of the WHO Classification of Tumors series https://publications.iarc.who.int/Book-And-Report-Series/Who-Classification-Of-Tumours/Central-Nervous-System-Tumours-2021, accessed on 21 May 2026), published in 2021. This framework integrates histology, immunohistochemistry, and molecular testing. The increasing impact of molecular diagnostics has led to significant changes in the classification, nomenclature, and grading of CNS tumors [12].

The nomenclature of these tumors takes into account multiple factors, including tumor cell origin, specific histological and molecular features, age, anatomical location, tumor grade, and molecular alterations. Traditionally, CNS tumor grading differed from that of other neoplasms, as it was applied across different tumor entities and correlated with an idealized clinical-biological behavior.

In the updated edition, the classification has shifted toward a within-type grading system.

This approach aligns more closely with grading systems used for non-CNS tumors, allowing greater flexibility in assigning grades relative to tumor type and better reflecting underlying biological behavior rather than clinical outcomes, e.g., overall survival, which are influenced by treatment. For example, there is neither grade 1 IDH mutant astrocytoma nor oligodendroglioma, nor grade 4 meningioma [13].

### 2.2. Immunohistochemical Markers

Immunohistochemistry (IHC) is a cornerstone of standard diagnostic pathology, acting as an indispensable bridge between histology and molecular biology. Beyond its established role in the diagnosis of CNS tumors, IHC also provides critical information for prognostic evaluation, survival prediction, and assessment of therapeutic response. By utilizing specific antibodies and biomarkers, IHC enables the characterization of tumor differentiation, maturation, and progression. Key IHC markers commonly used in the evaluation of CNS neoplasms are summarized in Table 1.

Despite its central diagnostic role, IHC has important limitations in CNS tumor diagnostics, particularly in poorly differentiated or highly heterogeneous tumors. Interpretation of immunostaining is often influenced by staining intensity, distribution, fixation quality, and the absence of universally standardized positivity thresholds for several markers [14]. Moreover, many commonly used markers exhibit limited specificity and may show overlapping expression across distinct tumor entities. For example, Glial fibrillary acidic protein (GFAP), although considered a classical glial marker, may also be expressed in reactive astrocytes, ependymal tumors, schwannomas, and certain metastatic neoplasms, potentially complicating the distinction between reactive and neoplastic processes. Similarly, S-100 protein demonstrates broad expression across glial, Schwannian, melanocytic, and mesenchymal tumors, limiting its standalone diagnostic utility. Consequently, contemporary neuropathology increasingly relies on integrated diagnostic approaches combining histomorphology, immunophenotyping, molecular profiling, and methylome-based classification rather than on isolated marker interpretation.

GFAP is a cytoplasmic intermediate filament protein that is positive in most astrocytic tumors and is widely used to distinguish them from non-glial neoplasms [15]. However, GFAP expression is not limited to neoplastic astrocytes; it can also be observed in reactive and normal astrocytes, as well as in neoplastic and non-neoplastic oligodendrocytes and ependymal cells [16]. In gliosarcoma, the glial component is GFAP positive, whereas the sarcomatous component is rich in reticulin. Moreover, in ganglioglioma, the ganglionic component expresses neuronal markers. Notably, GFAP expression tends to decrease with increasing tumor grade, reflecting poorer differentiation of the tumor [15].

Additional immunohistochemical markers may provide diagnostic value in specific tumor subtypes. In diffuse gliomas, oligodendrocyte transcription factor 2 (Olig2) supports glial differentiation but is not specific for oligodendroglioma. By contrast, α-thalassemia/mental retardation syndrome X-linked (ATRX) loss is typically associated with astrocytic lineage, whereas retained ATRX expression is generally expected in oligodendroglioma in the appropriate molecular context. In ependymomas, typical markers include vimentin, S-100 protein, synaptophysin, focal cytokeratin (CK), and epithelial membrane antigen (EMA), often showing a characteristic perinuclear dot-like pattern. Myxopapillary ependymoma represents a distinct subtype with a peculiar histomorphology and a typical location, almost exclusively, in the filum terminale and conus medullaris. It is usually EMA-negative, while GFAP positivity helps to exclude other tumors that may typically arise in this region, such as chordoma, chondrosarcoma, or paraganglioma. Importantly, GFAP is not completely specific for glial cells, as it may be expressed in schwannomas, choroid plexus tumors, and certain tumors of salivary and sweat glands [17]. Diagnostic pitfalls may arise in tumors with atypical immunophenotypes. For instance, GBMs with reduced or absent GFAP expression may be misinterpreted as metastatic carcinoma or sarcoma, particularly in small biopsy samples. Conversely, focal cytokeratin or EMA expression in high-grade gliomas and ependymomas may mimic metastatic epithelial tumors. Similarly, loss of ATRX expression is highly supportive of astrocytic lineage; however, technical artifacts and subclonal loss patterns may complicate interpretation. These limitations underscore the importance of correlating immunohistochemical findings with morphology, radiological features, and molecular analyses.

Vimentin is another cytoplasmic intermediate filament protein, but it is highly non-specific. It is expressed in a wide range of mesenchymal origin, mesenchymal and epithelial tumors, developing neurons, and glial tumors. In astrocytomas, vimentin shows strong positivity, mostly in an inverse relationship with GFAP, with the highest expression in high-grade tumors. Also, it is expressed in ependymomas and meningiomas, while it is typically negative or weakly positive in oligodendrogliomas [18].

CKs are intermediate filaments present in almost all epithelial cells and comprise at least 20 subtypes based on their molecular weight. While they are present in normal epithelium and epithelial tumors, variable expressions may also occur in numerous mesenchymal and other tumors. In CNS, CK positivity is uncommon, found mainly in choroid plexus tumors and certain meningioma subtypes. Their principal diagnostic value in neuropathology lies in differentiating primary CNS tumors from metastatic lesions [19].

Synaptophysin is a major transmembrane glycoprotein expressed in normal, reactive, and neoplastic neuroectodermal or neuroendocrine cells, making it a preferred marker for tumors of neuronal or neuroendocrine origin. During early neurogenesis, other markers such as β-tubulin may be expressed instead. In neuroendocrine tumors, such as paraganglioma, chromogranin A is also typically positive [17,20].

S-100 protein is a calcium-binding protein, originally isolated from the CNS. It is expressed in glial cells, Schwann cells, and melanocytes, and tumors derived from these lineages. It is also found in many other cell types, including chondrocytes, adipocytes, and myoepithelial cells, as well as their neoplastic counterparts [17,21]. S-100 positivity is also seen in Langerhans cell histiocytosis and may be present in small percentages in meningiomas, usually focally and with low intensity [22].

EMA is a glycoprotein considered a marker of normal and neoplastic epithelium and perineurium. Although primarily associated with epithelial differentiation, EMA expression has also been reported in various mesenchymal tumors, melanomas, and lymphomas. Within the CNS, EMA serves as a valuable diagnostic marker in the evaluation of meningiomas, ependymomas, chordomas, and metastatic carcinomas. In particular, EMA negativity is important for distinguishing schwannomas from solitary fibrous tumors, formerly termed hemangiopericytomas, which represent key histopathological mimickers of meningiomas. Additional differential diagnostic considerations include hemangioblastoma, which is typically EMA-negative, and metastatic renal cell carcinoma (RCC), which is generally EMA-positive. Despite their differing EMA expression profiles, these tumors may exhibit overlapping morphological characteristics and commonly express both vimentin and carbonic anhydrase IX (CA IX). However, several other immunohistochemical markers help distinguish them: hemangioblastoma shows positivity for vascular markers and stromal cell expression of α-inhibin, whereas renal cell carcinoma is positive for CK, paired box gene 8 (PAX8), and cluster of differentiation 10 (CD10) [13,17].

Leukocyte common antigen (LCA) does not differentiate between normal lymphocytes and lymphomas; therefore, a comprehensive IHC panel is required to determine B-cell, T-cell, or other lineage. Most primary CNS lymphomas are of B-cell origin. LCA is expressed in all leukocytes except plasma cells. Caution is required in LCA-negative lymphomas, such as plasmablastic and lymphoblastic lymphomas, anaplastic large cell lymphomas, and Reed-Sternberg cells in classic Hodgkin’s lymphomas [17].

Human melanoma black 45 (HMB-45) is a specific marker that is diffusely positive in primary melanocytic tumors from leptomeningeal melanocytes, as well as metastatic melanoma, which is the most frequent melanoma type in the CNS. Another specific marker is Melan A., whereas S-100 expression is typically elevated [17].

The IHC panel for CNS germ cell tumors is similar to that used for germ cell tumors in other anatomical locations. It does not help distinguish primary CNS from metastatic origin. Markers include placental alkaline phosphatase (PLAP), α-fetoprotein (AFP), human chorionic gonadotropin (HCG), cluster of differentiation 30 or 117 (CD30, CD117), and others, allowing diagnosis of germinoma, embryonal carcinoma, yolk sac tumor, choriocarcinoma, and teratoma [17].

Ki-67 is used to assess tumor proliferative activity. It correlates with the prognosis, patient survival, and tumor grade. It is expressed during all active phases of the cell cycle [23,24]. In contrast, phosphohistone H3 (PHH3) occurs exclusively during mitosis. Another method of assessment is counting mitotic figures on hematoxylin-eosin staining [25].

Finally, p53 is a protein encoded by the tumor suppressor gene TP53 and plays a critical role in maintaining genomic stability. Positive p53 expression has been associated with poorer clinical outcomes, including reduced survival and accelerated progression toward higher-grade lesions [26].

Although several IHC markers remain indispensable in routine neuropathology due to their accessibility, rapid turnaround time, and cost-effectiveness, increasing diagnostic complexity has accelerated the transition toward molecularly integrated classification systems. Markers such as IDH1 R132H, ATRX, and H3K27M retain major practical importance because they correlate strongly with biologically defined tumor entities and can serve as reliable surrogates for molecular alterations. In contrast, less specific lineage-associated markers, including S-100, vimentin, and neuron-specific enolase (NSE), are progressively losing their independent diagnostic value owing to considerable overlap in expression patterns across different tumor types. Furthermore, methylome profiling and next-generation sequencing increasingly outperform conventional IHC in diagnostically ambiguous tumors, particularly in pediatric and poorly differentiated CNS neoplasms, where morphology and immunophenotype alone may be insufficient for accurate classification. Biomarker utility should always be correlated with multi-omics profiling and computational diagnostic consensus to address spatial intratumoral heterogeneity.

## 3. Genetic Alterations of Brain Tumors

### 3.1. Integrated Molecular Diagnosis

While histopathological examination and IHC remain fundamental components of CNS tumor diagnostics, the 2021 WHO Classification introduced an integrated diagnostic approach that combines histological, immunohistochemical, and molecular findings. This shift reflects the growing recognition that tumors with similar histomorphological features may differ substantially in their molecular profile, biological behavior, prognosis, and therapeutic response. Consequently, specific molecular alterations are now considered defining diagnostic criteria for several CNS tumor entities and are increasingly used for prognostic stratification and treatment selection.

Brain tumors represent a group of neoplasms arising from uncontrolled cell proliferation within the brain or the CNS. Their pathogenesis is closely associated with genetic and epigenetic alterations that disrupt key regulatory mechanisms, including cell cycle control, proliferation, and DNA damage response. Such changes enable tumor cells to evade normal growth constraints and promote tumor development and progression [27].

Over the past few decades, advances in molecular biology have significantly improved our understanding of the genetic landscape of brain tumors. A significant proportion of these tumors harbor specific genetic mutations that determine their biological behavior, aggressiveness, and response to therapeutic interventions [28]. Consequently, in the 5th edition of the *WHO Classification of CNS Tumors*, molecular diagnostics play a crucial role in tumor classification, often superseding purely histological criteria. This approach has enabled the distinction between adult- and pediatric-type gliomas, the redefinition of tumor subclassification (e.g., ependymomas and medulloblastomas), and the identification of entities defined primarily by molecular features [13].

Several pediatric CNS tumor entities are now classified using DNA methylation profiling (“methylome-defined” tumors). DNA methylation profiling analyzes genome-wide epigenetic patterns and allows identification of biologically distinct tumor subgroups that may not be distinguishable by histology alone [29].

Key genetic alterations in CNS tumors are summarized in Table 2.

GBMs were among the first tumor types in which an epigenetic biomarker was successfully implemented in clinical practice [30,31]. DNA methylation profiling is essential or highly informative for the accurate classification of selected CNS tumor entities, particularly when histology and conventional molecular testing are insufficient, e.g., diffuse glioneuronal tumor with oligodendroglioma-like features and nuclear clusters, diffuse pediatric type-high-grade glioma H3-wildtype and IDH-wildtype, posterior fossa ependymomas or medulloblastomas, non-WNT/non-SHH. In addition, loss of H3K27 trimethylation (H3K27me3), which can be detected bIHC, serves as a valuable diagnostic marker for distinguishing posterior fossa ependymomas from other ependymoma subtypes [32,33].

Among the most well-characterized genetic alterations are mutations in the IDH1 and IDH2 genes, which are particularly associated with certain types of gliomas and are often linked to better prognosis. Amplification and overexpression of the EGFR gene play a crucial role in promoting tumor cell proliferation and survival, especially in more aggressive tumor forms. Mutations in the tumor suppressor gene TP53 lead to impaired control of the cell cycle and reduced ability to initiate apoptosis in response to genomic damage. In addition to these genetic changes, epigenetic modifications such as methylation of the O-6-methylguanine DNA methyltransferase (MGMT) gene promoter are of great clinical importance, as they can influence the tumor’s sensitivity to alkylating chemotherapeutic agents [34]. Furthermore, the BRAF V600E mutation is frequently observed in pleomorphic xanthoastrocytoma, often accompanied by positive BRAF immunoreactivity and CDKN2A deletion. TERT promoter mutations also represent recurrent molecular alterations across several CNS tumor subtypes. In contrast, pilocytic astrocytoma is commonly characterized by the presence of a KIAA1549–BRAF fusion, typically in the absence of detectable BRAF V600E immunoreactivity. Importantly, the diagnostic and prognostic utility of these biomarkers should be interpreted within the broader framework of multi-omics profiling and integrated computational diagnostic approaches to account for spatial intratumoral heterogeneity [35].

Some newly recognized entities, based on molecular findings, include pediatric tumors such as diffuse astrocytoma, diffuse low-grade glioma, diffuse midline glioma, diffuse hemispheric glioma, and diffuse pediatric-type high-grade glioma. Molecular-driven subclassification is used for entities previously known as ependymomas or medulloblastomas [13].

Among the most clinically relevant molecular markers in CNS tumors are IDH1/2 mutations, 1p/19q codeletion, EGFR amplification, TP53 alterations, and MGMT promoter methylation, each contributing to tumor classification, prognostic evaluation, and therapeutic decision-making.

### 3.2. IDH1/2 Mutations

According to the WHO 2021 classification, IDH mutation status is a key molecular criterion for classifying diffuse gliomas. Specifically, astrocytomas are defined by the presence of IDH mutations in the absence of 1p/19q codeletion, whereas oligodendrogliomas are characterized by the coexistence of IDH mutations and 1p/19q codeletion.

DHs comprise three isoenzymes, IDH1, IDH2, and IDH3, which catalyze oxidative decarboxylation reactions within the Krebs cycle [36]. IDH1 and IDH2 are located on chromosomes 2 and 15, respectively, and encode NADP^+^-dependent homodimeric enzymes sharing approximately 70% sequence identity. In contrast, IDH3 is a structurally distinct, mitochondrial NAD^+^-dependent heterotetramer that catalyzes an irreversible step of the tricarboxylic acid (TCA) cycle. Importantly, the oncogenic neomorphic mutations relevant to glioma biology predominantly involve IDH1 and, less frequently, IDH2, rather than IDH3 [37]. In terms of subcellular localization, IDH1 is found in the cytoplasm and peroxisomes, while IDH2 and IDH3 are localized within mitochondria [38]. Functionally, IDH1 and IDH2 catalyze the reversible oxidative decarboxylation of isocitrate to 2-oxoglutarate (2OG) in a reaction dependent on oxidized nicotinamide adenine dinucleotide phosphate (NADP^+^), while simultaneously producing reduced nicotinamide adenine dinucleotide phosphate (NADPH), which is essential for maintaining cellular redox balance. In contrast, IDH3 catalyzes the nicotinamide adenine dinucleotide (NAD+)-dependent conversion of isocitrate to 2-oxoglutarate within the tricarboxylic acid (TCA) cycle, a reaction that is regarded as irreversible under physiological conditions [37]. Mutations in IDH1 and, less frequently, IDH2 were first identified in GBMs through exome-wide sequencing studies in 2008 [39]. These mutations are highly prevalent in lower-grade gliomas, occurring in approximately 80% of grade II and III astrocytomas and oligodendrogliomas, and are also present in previously termed “secondary” GBMs that develop from these tumors [40]. The most common mutations in IDH1 and IDH2 involve a single amino acid substitution, in which arginine is replaced, leading to a neomorphic enzymatic activity. As a result, the normal product α-ketoglutarate (α-KG) is converted into D-2-hydroxyglutarate (D-2HG), an oncometabolite implicated in tumor biology. D-2HG has also emerged as a promising biomarker for monitoring therapeutic response [41]. In IDH-mutant tumors, intracellular D-2HG concentrations may reach levels ranging from 1 to 30 mM [42]. Under physiological conditions, D-2HG levels are tightly regulated, partly through transport mechanisms such as the citrate transporter (CTP/CIC). However, its accumulation in mutant cells is thought to contribute to tumorigenesis, although the exact molecular mechanisms remain not fully elucidated [38].

Due to its structural similarity to α-ketoglutarate (α-KG), D-2HG competitively inhibits multiple α-KG-dependent dioxygenases, including ten-eleven translocation (TET) DNA demethylases and Jumonji-C domain-containing histone demethylases [43]. This inhibition results in widespread epigenetic dysregulation characterized by DNA and histone hypermethylation, impaired cellular differentiation, and establishment of the glioma CpG island methylator phenotype (G-CIMP). In addition, altered dioxygenase activity affects hypoxia signaling, chromatin organization, and metabolic adaptation, thereby contributing to gliomagenesis and tumor progression [44].

IDH1/2, ATRX, and 1p/19q co-deletion are important for the division of gliomas into three groups. IDH1 mutations are present in most astrocytomas, oligodendrogliomas, and previously termed “secondary GBM”, but absent in “primary” GBM and ependymomas. Immunohistochemical staining for IDH1 is commonly used as an initial screening approach for detecting IDH alterations; however, because immunonegativity does not exclude the presence of less common IDH variants, additional molecular analyses may be required. Beyond IDH status, ATRX mutations represent another important molecular feature and are typically associated with loss of ATRX protein expression in tumor cells. Diffuse gliomas are currently classified according to the combined assessment of IDH mutation status, ATRX expression, and 1p/19q codeletion. Tumors harboring IDH mutation and 1p/19q codeletion are classified as oligodendrogliomas, whereas IDH-mutant tumors lacking 1p/19q codeletion, frequently associated with ATRX loss, are classified as astrocytomas. In contrast, diffuse astrocytic tumors with IDH-wildtype status that exhibit specific molecular features, including EGFR amplification, TERT promoter mutations, or the combined gain of chromosome 7 and loss of chromosome 10, are classified as IDH-wildtype glioblastomas [13].

### 3.3. EGFR Amplification

The EGFR gene, located on chromosome 7, encodes a transmembrane glycoprotein belonging to the receptor tyrosine kinase (RTK) family [45]. EGFR acts as a pivotal modulator of diverse intracellular signaling networks that regulate essential cellular functions, including proliferation, motility, survival, and neoplastic transformation. In normal physiology, EGFR signaling is strictly growth-factor-dependent. However, oncogenic alterations, such as point mutations or aberrant expression of receptor isoforms, can induce constitutive, ligand-independent activation, thereby facilitating tumorigenic processes [36]. In brain tumors, one of the most extensively studied EGFR alterations is the EGFR transcript variant III (EGFRvIII) [46]. EGFRvIII arises from genomic rearrangements associated with EGFR gene amplification and represents a tumor-specific receptor variant. Notably, its expression is restricted to GBMs and other malignant cells, while being absent in normal tissues, highlighting its potential diagnostic and therapeutic relevance [47]. EGFR gene amplification (EGFR Amp) is observed in approximately two-thirds of GBM, with roughly half of these cases also harboring EGFRvIII or single-nucleotide variants. This amplification leads to overexpression of the EGFR protein, thereby driving tumor cell proliferation, angiogenesis, and invasion through activation of the rat sarcoma (RAS) and phosphatidylinositol 3-kinase/protein kinase B (PI3K/AKT) signaling pathways. Moreover, EGFR Amp-associated genomic rearrangements increase the frequency of EGFRvIII expression, which can initiate downstream signaling independently of extracellular ligands, further contributing to tumorigenic progression [45]. Despite initial sensitivity to RTK inhibitors, tumors harboring EGFR Amp often develop therapeutic resistance, as evidenced by clinical observations [48]. Due to their high prevalence and biological relevance, EGFR mutations and amplifications are considered important prognostic biomarkers in GBM [49].

### 3.4. TP53 Mutations

TP53 is a pivotal tumor suppressor gene located on chromosome 17 that encodes the nuclear transcription factor p53 [28], frequently described as the “guardian of the genome”. p53 regulates cellular responses to diverse stressors, including DNA damage, oncogene activation, nutrient deprivation, and hypoxic conditions [50,51]. Beyond its canonical role in maintaining genomic integrity, p53 regulates metabolism, stemness, autophagy, invasion, metastasis, interactions with the tumor microenvironment (TME), and immune responses [52]. Emerging evidence further indicates that the functions of p53 extend well beyond its classical roles in cell-cycle regulation and apoptosis, highlighting its pleiotropic effects in tumor biology [53]. In CNS tumors, p53 participates in remodeling the TME by modulating inflammatory cytokines, immune signaling, angiogenesis, and metabolic adaptation. Mutant TP53 may promote immunosuppressive tumor states and enhance glioma progression through interactions with hypoxia-related and pro-inflammatory pathways [54]. In addition, p53 has emerged as an important regulator of epitranscriptomic processes, including N6-methyladenosine (m6A) RNA modification pathways, which influence mRNA stability, stemness, and therapeutic resistance in cancer cells [55,56]. Another rapidly developing area is the role of p53 in ferroptosis, an iron-dependent form of regulated cell death associated with lipid peroxidation. Depending on the cellular context, p53 may promote or suppress ferroptosis by regulating targets such as SLC7A11, thereby influencing tumor survival, oxidative stress responses, and sensitivity to therapy. These non-canonical functions further highlight the central role of TP53 alterations in CNS tumor progression and therapeutic resistance [57,58,59].

Most TP53 alterations are missense mutations. In addition to the loss of their normal tumor-suppressive function, these TP53 mutants often acquire novel oncogenic properties that contribute to the development of malignant traits in cancer cells [60]. TP53 point mutations are significantly more prevalent in secondary brain tumors, occurring in approximately 90% of cases, compared to 30% in primary tumors, and may be absent in some primary lesions [61]. One proposed mechanism of tumor promotion involves dysregulation of the mevalonate (MVA) pathway [62].

### 3.5. MGMT Promoter Methylation

The MGMT gene, located on chromosome 10 [36], encodes a DNA repair enzyme that reverses DNA alkylation by removing alkyl groups from guanine, thereby preventing DNA damage-induced apoptosis [48]. MGMT promoter methylation represents one of the most clinically srelevant predictive biomarkers in GBM, IDH-wildtype, as reduced MGMT expression limits repair of temozolomide-induced O6-methylguanine lesions and is generally associated with increased sensitivity to alkylating chemotherapeutic agents, particularly temozolomide (TMZ) [28]. 

MGMT promoter methylation is detected in approximately 40% of GBMs. Historical studies using the former primary/secondary GBM terminology reported higher methylation frequencies in tumors previously termed secondary GBMs than in primary ones, with frequencies of approximately 75% and 36%, respectively [36]. These findings should be interpreted in light of the current WHO CNS5 classification, in which GBM is restricted to IDH-wildtype tumors, whereas many lesions formerly referred to as secondary GBMs are now classified as astrocytoma, IDH-mutant, CNS WHO grade 4, when current diagnostic criteria are met.

In normal tissue, most CpG sites within the MGMT promoter region remain unmethylated. In tumor cells, cytosine residues at these CpG sites are often methylated, increasing the binding affinity of proteins such as methyl-CpG-binding protein 2 (MeCP2) and methyl-CpG-binding domain protein 2 (MBD2). These proteins modify chromatin structure, inhibit transcription factor binding, and effectively silence MGMT expression [63].

Paradoxically, reduced MGMT expression enhances treatment efficacy, as tumor cells become less capable of repairing chemotherapy-induced DNA damage. Consequently, patients with MGMT promoter methylation show improved response to TMZ, making its assessment a critical component of clinical decision-making [64,65].

## 4. Epigenetic Alterations of Brain Tumors

Epigenetic regulation refers to heritable and reversible changes in gene expression that occur without alterations in the underlying DNA sequence. These mechanisms include DNA methylation, histone modifications, chromatin remodeling, enhancer regulation, and non-coding RNAs. Epigenetic regulation involves multiple mechanisms beyond histone modifications, including DNA methylation, alterations in chromatin architecture, long non-coding RNAs (lncRNAs), enhancer activity, and microRNAs (miRNAs). These epigenetic modifications can be stably transmitted through successive cell divisions, thereby contributing to the maintenance of cellular identity and function. Furthermore, cell signaling pathways, together with extracellular stimuli, can dynamically influence the epigenome, which regulates diverse biological processes through enzymes that modify transcription factors, DNA, and histones, as well as through the actions of non-coding RNAs [66]. Patients suffering from cancer based on different epigenetic profiles may have different manifestations and survival outcomes despite the same grade and stage of the disease. Epigenetic heterogeneity occurs at the cellular level, and each cell in a tumor may exhibit distinct epigenetic patterns, whether in specific genes, the genome as a whole, or histones [67]. The interplay among multiple epigenetic mechanisms may collectively contribute to tumor initiation and progression [66]. Dysregulated epigenomes (Table 3), including histone modifications, disorganized chromatin structure, DNA methylation, and non-coding RNAs, have been observed in brain tumors in adults and children over the past two decades [68,69].

### 4.1. DNA Methylation

Modification or repression of transcription is mediated by an epigenetic mechanism, DNA methylation, in which methyltransferases add methyl groups to promoter regions or CpG repeat sequences. As a stable epigenetic modification maintained through DNA replication, DNA methylation plays a critical role in regulating cellular processes, including differentiation, proliferation, and tumor initiation and progression. However, its effects can be reversed by DNA demethylation. A common epigenetic feature of cancer is promoter hypermethylation, which may silence tumor suppressor genes, TEN-ELEV together with global hypomethylation, which may contribute to genomic instability and aberrant oncogene activation. [70]. Oncogene expression occurs through methylation near enhancer regions, and tumor suppressor genes are repressed through methylation of CpG-rich sequences located within promoter regions. Both mechanisms enhance tumor growth [71]. Methylation typically occurs in CpG-rich regions of DNA with histones that are both hypoacetylated and hypermethylated [70]. An instance of this is methylation involving mutated IDH1/2 and, in the case of ependymomas, the development of the CpG island methylator phenotype (CIMP) [68]. Accumulation of D-2-hydroxyglutarate (D2HG; a defective product of the Krebs cycle) leads to a metabolic change initiated by the mutant enzyme IDH, which is frequently found in both children and adults in gliomas [72]. The accumulation of D-2-hydroxyglutarate creates an environment in which multiple mutations can arise later [73]. D-2HG exerts its oncogenic effects by inhibiting the Ten-eleven translocation (TET) DNA demethylases and Jumonji-C domain-containing histone demethylases. This inhibition promotes widespread promoter hypermethylation, thereby highlighting the central role of IDH mutations in driving tumor transformation and epigenetic reprogramming. [72]. IDH1 mutations occur in diffuse gliomas in children, and H3K36 and H3K27 methylation are also involved in the progression from low-grade gliomas to previously termed “secondary” GBMs [74]. Stable methylation patterns in ependymal cells suggest that they serve to assign them to cell lineages rather than to tumor progression [75]. Tumor treatment strategies involve inactivating IDH or blocking D2HG. GBM (GBM xenografts) growth was slowed after treating mice with DNA methylation inhibitors that had an IDH mutation [76]. Clinical studies have shown favorable results, especially with lower-grade tumors [77]. Preclinical studies have also demonstrated the therapeutic potential of IDH mutation-targeted immunotherapy in glioma treatment, either in combination with radiotherapy or together with TMZ [73,78]. Furthermore, additional mechanisms have been proposed involving metabolic and apoptotic pathways altered in IDH-mutant cells, although these remain to be fully elucidated [73,79]. Among epigenetic biomarkers, DNA methylation of the MGMT gene has emerged as a clinically important predictor of treatment response in GBM. When the MGMT promoter is unmethylated, MGMT expression is generally maintained, enabling tumor cells to repair TMZ-induced O6-methylguanine lesions and thereby contributing to treatment resistance. Conversely, promoter methylation reduces MGMT expression and is associated with improved response to TMZ. 

Methylated promoter regions are found in only 30% of patients who respond favorably to chemotherapy, e.g., TMZ, compared to patients who have unmethylated promoter regions [80]. Clinical treatment (phase I/II) via glycogen synthase kinase 3β inhibition together with TMZ enhanced the effect of TMZ without adverse side effects and increased survival in unmethylated MGMT-related promoter regions [81]. In another study, the increase in TMZ through inhibition of glycogen synthase kinase 3β was based on cytosine methylation in the promoter region of the MGMT gene [82].

### 4.2. Histone Modifications

The main histone modifications include demethylation/methylation and deacetylation/acetylation [71], as well as phosphorylation and ADP ribosylation [83], which can be activating/deactivating in relation to gene expression [71]. Less common histone modifications, e.g., in GBM, are lactylation, succinylation, and crotonylation [84]. Histone modifications primarily occur at the N-terminal tails or within the core domains of histone proteins. Histone methylation can either activate or repress gene expression depending on the specific amino acid residue involved and the degree of methylation, typically mediated by methyltransferases through the addition of one, two, or three methyl groups to lysine or arginine residues. Importantly, this process is reversible and can be regulated by histone demethylases [70]. The most well-known are histone modifications on lysine residues in the N-terminal ends of both H4 and H3 [85]. In cancer cells, a tight chromatin configuration is created by the loss of histone marks of tumor suppressor genes and by the relaxation of chromatin conformation after the loss of repetitive regions or suppressor marks in subtelomeric DNA [83]. In approximately 80% of patients with diffuse midline glioma and in 20% of pediatric patients with GBM, this epigenetic alteration is driven by an H3 K27M mutation, resulting from a a lysine-to-methionine substitution at residue 27 of histone H3 or H3.3. The mutant histone interferes with Polycomb repressive complex 2 (PRC2) activity, leading to widespread loss of H3K27me3 and extensive transcriptional reprogramming.

The H3K27M oncohistone inhibits PRC2 enzymatic activity, leading to a global reduction in H3K27 trimethylation and profound transcriptional reprogramming [86]. The H3K27M oncohistone inhibits PRC2 enzymatic activity, leading to a global reduction in H3K27 trimethylation and profound transcriptional reprogramming. Loss of H3K27 trimethylation (H3K27me3) can also occur in posterior fossa group A ependymomas through mechanisms that are frequently independent of H3K27M mutations [87]. This epigenetic alteration contributes to aberrant transcriptional regulation and may promote oncogene activation [86]. In addition, histone H3 lysine demethylases are also expressed in GBM [31]. Interestingly, diffuse midline glioma is dependent on PRC2 activity for growth despite the aforementioned 2 inhibition [88]. However, in medulloblastoma, PRC2-1 expression is increased [89]. The H3 variant is important in this, where H3K27M occurs with PI3K (phosphoinositide 3-kinase) and with ACVR1 (activin-receptor type 1) and H3.3.K27M, which is less differentiated, more aggressive, and occurs with TP53 deletions [90,91]. Preclinical and clinical investigations evaluating PRC2/enhancer of zeste homolog 2 (EZH2) inhibitors, such as tazemetostat, are currently underway for the treatment of medulloblastoma, GBM, and ependymoma [66]. 

On 6 August 2025, the U.S. Food and Drug Administration (FDA) granted accelerated approval to dordaviprone, an oral imipridone and protease activator, for adult and pediatric patients aged 1 year and older with diffuse midline glioma harboring an H3K27M mutantion and progressive disease following prior therapy. This approval was based on an integrated efficacy population of 50 patients enrolled across five open-label, non-randomized trials, with an overall response rate of 22% and a median duration of response of 10.3 months. Therefore, dordaviprone should be discussed as a recently approved targeted systemic option for a molecularly defined and recurrent/progressive disease setting, rather than as a broadly effective treatment for all histone-mutant CNS tumors [92]. Specifically, dordaviprone acts through a dual mechanism, since it is an allosteric activator of the mitochondrial caseinolytic protease P (ClpP) and a selective antagonist of the dopamine receptor D2, thereby triggering a stress response, promoting apoptosis and cell cycle arrest, or exerting antiproliferative effects [92]. Dordaviprone was approved for recurrent H3K27M-mutant diffuse midline glioma because these tumors have unique epigenetic and metabolic vulnerabilities. Although other tumors also carry histone mutations, they differ biologically and may not depend on the same pathways targeted by the drug, such as mitochondrial stress responses, ClpP activation, or DRD2 signaling [92,93,94]. Therefore, histone mutations alone do not predict sensitivity. Toxicity data from clinical studies showed mainly manageable adverse effects, including fatigue, nausea, headache, vomiting, lymphopenia, elevated liver enzymes, and QTc prolongation. Compared with conventional chemotherapy, dordaviprone appears relatively well tolerated, with limited severe hematologic or neurologic toxicity [92,95].

Treatment also takes place with poly ADP-ribose polymerase inhibitors, which participate in the repair of DNA breaks and chromatin remodeling through histone modifications. Olaparib combined with TMZ was favorably tolerated (phase I). Other histone modifications affecting gene expression are acetylation and bromodomain and extraterminal proteins, for which treatment consists of deacetylase inhibitors and inhibitors of the given proteins [66].

### 4.3. miRNA Regulation

Additional epigenetic regulators implicated in tumor biology include non-coding RNAs, particularly miRNAs and lncRNAs. Most miRNAs are transcribed from independent genomic loci and subsequently undergo a multistep maturation process involving the generation of precursor miRNAs before becoming functionally active [96]. These molecules regulate gene expression primarily through post-transcriptional modulation of mRNA translation and stability [97]. Depending on their biological context and target genes, miRNAs may function either as oncogenes or as tumor suppressors during tumor development and progression [66]. MiRNAs regulate DNA methylation through DNA methyltransferases (DNMTs) or methylation-related proteins [98]. Under non-pathological conditions, they participate in apoptosis, angiogenesis, cell cycle, and DNA repair [66]. In adult brain tumors, several tumor-suppressive miRNAs are downregulated as tumor suppressors. Examples in GBM include miR-124, miR-138, miR-7, miR-181a/b, miR-128, miR-137 [99,100]. The accumulation of methyl groups in the promoter region suppresses the expression of, e.g., miR-410 in GBM, which could prevent cell division [101]. Targeting DNMTs via miR-185 resulted in the reversal of DNA methylation and the methylation of hypermethylated genes in GBM. Similarly, miR-101 reduced histone methylation, targeting DNMT3A, Embryonic Ectoderm Development (EED), and EZH2 in GBM to reactivate repressed (hypomethylated) genes [102].

Conversely, selected miRNAs, such as miR-21 in GBM [103], may function as oncogenic miRNAs and contribute to invasion, proliferation, drug resistance, and cell survival [104]. In addition to miR-21, miR-155, and miR-34a are accompanied by hypomethylation [105]. Tumor suppressor miRNAs such as miR-125b, miR-218, and miR-1253 have been observed in pediatric patients with diffuse midline glioma and medulloblastoma [106]. Histone deacetylation is also associated with the suppression of miRNA genes [105]. Therapeutic strategies involving miRNAs primarily focus on either restoring the expression of tumor suppressor miRNAs or inhibiting oncogenic miRNAs. Various delivery approaches have been explored to optimize therapeutic efficacy. Locoregional administration can bypass the BBB, thereby enhancing target-site delivery, although this approach is inherently more invasive. Alternatively, systemic administration utilizes both viral and non-viral delivery systems. Viral vectors, including adenoviruses and retroviruses, offer efficient gene delivery, whereas non-viral platforms, typically based on polymers and lipid-derived carriers, are generally considered safer and exhibit improved biocompatibility [106]. Tumor suppressors are restored using miRNA mimetics (single- or double-stranded synthetic RNAs) that functionally mimic endogenous miRNAs, or an indirect method using a viral vector that subsequently expresses the miRNA. Oncogenic miRNAs are inhibited using a non-viral method using antagomirs or antisense oligonucleotides that bind to miRNAs and cause degradation of mature miRNAs [107].

## 5. Future Trends in Diagnosis and Treatment

### 5.1. CNS Tumor Heterogeneity, Therapeutic Resistance, and Clinical Challenges

Tumor heterogeneity represents a key consequence of the genetic and epigenetic alterations described above, and a major determinant of tumor behavior and therapeutic response. This heterogeneity refers to the diversity within cancers. It manifests at multiple levels, including intertumoral variability, observed between tumors of the same histological type in different patients, and intratumoral heterogeneity, which reflects the coexistence of diverse cancer cell populations within a single tumor mass. Additional complexity arises from differences between primary tumors and their corresponding metastases [108]. Altogether, cancer heterogeneity encompasses spatial variations across tumor regions and anatomical sites (spatial heterogeneity), as well as temporal changes (temporal heterogeneity) that occur during disease progression and under therapeutic pressure [109]. A major heterogeneity driver is the continuous process of clonal evolution since tumor progression can be considered as a Darwinian process that may follow different trajectories, including linear evolution, in which successive mutations accumulate within a dominant clone, and branched evolution, where multiple subclones diverge and coexist in parallel. In GBM, representative of the most heterogeneous CNS tumor, a prominence of branched evolution emerges and contributes to the coexistence of genetically and epigenetically distinct cellular subpopulations. In general, all the different cell subpopulations within the tumor are subjected to selective pressures imposed by TME and by therapeutic interventions. The TME involves a series of interconnected changes in the cellular landscape, spatial organization, and extracellular matrix composition [110]. However, in the context of CNS tumors, the TME exhibits distinctive characteristics compared with other tumor types and is therefore referred to as the neuro-TME. This highly specialized milieu comprises resident cellular populations, including microglia, astrocytes, and neurons, which actively interact with tumor cells and contribute to tumor development and disease progression. Moreover, unlike peripheral tissues, the CNS is endowed with a distinctive immunosuppressive landscape that significantly limits anticancer immune response. Neuro-TME also implies unique metabolic constraints, such as high glucose consumption, tight regulation of oxygen availability, and dependence on lactate and ketone bodies, that sustain growth and adaptation of cancer cells to harsh conditions, such as hypoxia, further contributing to therapeutic resistance [111]. Intratumoral heterogeneity in CNS tumors has important diagnostic and therapeutic implications and may significantly contribute to treatment failure. In GBM, tissue biopsies are highly invasive and often capture only a spatially limited representation of the entire lesion. Accordingly, multiregional analyses have demonstrated that spatially separated regions within the same tumor can exhibit distinct molecular and transcriptional profiles [112,113].

Notably, distinct regions within the same GBM tumor may correspond to different transcriptional subtypes, underscoring the limitation of single-site biopsy in capturing the full complexity of the lesion [114]. However, repeated tissue sampling is rarely feasible due to its invasive nature. In this context, liquid biopsy represents a promising alternative, as it may allow longitudinal monitoring of tumor-derived material in plasma or cerebrospinal fluid (CSF). However, its clinical implementation in GBM remains challenging [115].

In this context, the marked spatial, temporal, and cellular heterogeneity of GBM highlights the need for diagnostic strategies that go beyond single-sample characterization. The 2021 WHO classification remains the essential diagnostic framework for CNS tumors; however, its application to GBM may benefit from complementary multiregional, spatial, single-cell, and longitudinal analyses able to capture intratumoral diversity and tumor evolution. Thus, the WHO-based classification should not be considered obsolete, but rather an evolving system to be progressively refined.

In the end, this complex process results in the expansion of clones with enhanced fitness, promoting survival, invasion, and metastatic dissemination, including processes such as epithelial–mesenchymal transition (EMT). However, in the context of CNS tumors, a more relevant process is the proneural-to-mesenchymal transition (PMT), which consists of a transcriptional reprogramming at the basis of aggressiveness, invasiveness, and therapy-resistant phenotype development. Furthermore, PMT has often been associated with inflammatory signaling, hypoxia, and treatment-induced stress [116].

Consequently, tumor evolution underlies both disease progression and therapeutic resistance. Thus, the above-discussed genetic alterations, along with epigenetic plasticity, contribute to tumor heterogeneity by enabling reversible transitions between distinct cellular states dynamically. This tumor adaptability supports the coexistence of multiple tumor cell populations with different molecular and functional properties [116].

Thus, within this heterogeneous landscape, tumors harbor distinct cellular subpopulations, including a small but functionally critical fraction of cancer stem cells (CSCs), also referred to as tumor-initiating cells (TICs). These cells are characterized by self-renewal capacity, tumor-initiating potential, and the ability to generate phenotypically diverse progenies. Importantly, increasing evidence supports a high degree of cellular plasticity, whereby non-stem cancer cells can reacquire stem-like properties in response to TME cues, such as hypoxia or therapeutic stress. In GBM, CSCs, known as glioma-like stem cells (GSCs), are often associated with specific niches, including perivascular and hypoxic regions, and are commonly identified by markers such as CD133; however, no universal marker has been identified. Generally, CSCs are reported to be implicated in tumor maintenance, metastatic dissemination, and therapy resistance. Their relative quiescence, efficient DNA repair mechanisms, and activation of pro-survival signaling pathways make them key contributors to tumor relapse. The origin and maintenance of CSCs are governed by two not mutually exclusive models that likely coexist: the stochastic model, based on the accumulation of mutations across multiple clones, and the hierarchical model, in which CSCs occupy the apex of a cellular hierarchy while generating more differentiated progeny. The dynamic interplay between these models further contributes to tumor complexity and heterogeneity [117].

As regards therapeutic resistance, it can be broadly classified into intrinsic and acquired forms. Intrinsic resistance arises from pre-existing genetic and phenotypic features within tumor cells, whereas the acquired one develops over time under therapeutic pressure. The ability of tumors to develop resistance during treatment represents a major clinical challenge, as it not only compromises therapeutic efficacy and patient outcome but also actively contributes to the evolution of tumor heterogeneity. Specifically, during treatment, resistant clones may be positively selected, while less sensitive cells can enter transient drug-tolerant states and subsequently acquire stable resistance mechanisms. These processes involve additional genetic alterations, activation of alternative signaling pathways, and cellular reprogramming. Clinically, this dynamic often manifests as an initial therapeutic response followed by tumor relapse [117].

However, expanding knowledge on cancer heterogeneity serves to provide the molecular framework necessary to design targeted strategies. By identifying the unique vulnerabilities of treatment-resistant subpopulations, such as CSCs, researchers can transition from broad-spectrum approaches to precision therapies capable of eradicating the most resilient cellular niches.

However, GBM and other brain tumors present additional challenges that contribute to their highly aggressive behavior and poor prognosis. Among the most critical limitations is the scarcity of reliable and specific biomarkers for early and accurate diagnosis. Table 4 summarizes currently established and emerging diagnostic techniques for GBM, highlighting their advantages and limitations. A comprehensive review on this topic has been provided by Ronvaux et al. [118].

Unlike other solid tumors, GBM lacks well-established molecular signatures that can be easily detected through non-invasive methods, making rapid diagnosis particularly complex. However, to date, MGMT promoter methylation and IDH mutations have been identified as prognostic and predictive biomarkers in GBM. Also, circulating miRNAs, extracellular vesicles, and selected tumor-derived proteins are being investigated as minimally invasivebiomarkersfor disease diagnosis and monitoring. Other inherent challenges in GBM are associated with tissue biopsy. In fact, due to the complex brain anatomy, surgical biopsy procedures are invasive, carry significant risks, and are not always feasible, especially for tumors located in eloquent or deep brain regions. Moreover, the high tumor heterogeneity associated with it may make a single biopsy sample not an accurate representation of the full molecular landscape of the disease. As regards liquid biopsy, it faces significant limitations in GBM, mainly due to the presence of the BBB, which restricts the release of tumor-derived biomarkers, such as circulating tumor DNA (ctDNA), into the bloodstream, thereby reducing detection sensitivity. While CSFmay offer a more direct source of tumor biomarkers, its collection is still invasive and not routinely applicable for frequent monitoring. Again, it is not able to fully capture the spatial heterogeneity of the tumor, as the release of biomarkers is not homogeneous and can significantly vary across different tumor regions. The detection sensitivity has advanced thanks to ultrasensitive sequencing technologies and analysis of cell-free DNA fragmentation patterns, but their clinical use is still limited [118].

Thus, heterogeneity represents a central obstacle in CNS oncology due to its role in tumor adaptability, therapy resistance, and recurrences. Moreover, the associated lack of accessible and specific biomarkers, combined with the technical and biological challenges of both tissue and liquid biopsy, significantly delays diagnosis and significantly delays diagnosis and hinders timely therapeutic intervention. The framework that emerges is that understanding of the associated complexity is essential to guide the development of both diagnostic and therapeutic strategies that could be efficacious [119].

### 5.2. The Pharmacological Exclusion: P-gp and the Infiltrative Zone Resistance

The treatment of GBM is limited by a significant pharmacological shield that complements the tumor’s genetic complexity [120,121]. A critical clinical paradox exists: while contrast-enhanced magnetic resonance imaging (MRI) indicates a compromised and permeable Blood-Brain Tumor Barrier (BBTB) within the tumor core, the highly aggressive cells at the infiltrative edges remain protected by a functionally intact BBB [120,122,123]. This regional sequestration is primarily driven by the ATP-binding cassette transporter P-glycoprotein (P-gp/ABCB1), which often works in synergy with other efflux pumps such as the breast cancer resistance protein (BCRP/ABCG2 to restrict the entry of lipophilic small molecules [124].

Evidence suggests that P-gp expression is a dynamic survival mechanism rather than a constant feature [125,126]. Microenvironmental stress, particularly localized hypoxia, stabilizes the hypoxia-inducible factor 1 alpha (HIF-1α), which directly upregulates *ABCB1* expression to allow cellular detoxification under metabolic pressure [125,126]. This spatial heterogeneity results in a significant pharmacokinetic mismatch; therapeutic concentrations may be reached in the tumor core, yet sub-therapeutic levels at the outer zones may unintentionally promote the selection of resistant clones [124,127]. To address this active efflux, current trends move beyond early-generation P-gp inhibitors like *tariquidar* (which were limited by systemic toxicity) toward nanoparticle-based delivery systems. These advanced platforms aim to circumvent efflux by exploiting alternative transport pathways, such as receptor-mediated transcytosis, to ensure drugs reach their intended sites of action [124].

However, the consistent clinical failure of early-generation P-gp inhibitors suggests that targeting a single transporter is insufficient to overcome the multidrug resistance (MDR) phenotype. In our view, this intractability is sustained by a complex biological redundancy; when P-gp is blocked, compensatory upregulation of other ATP-binding cassette (ABC) transporters, such as BCRP, ensures continued cellular detoxification. Furthermore, the spatial heterogeneity of the BBTB means that global inhibition often leads to systemic toxicity before achieving therapeutic concentrations at the infiltrative edges. Therefore, the field must pivot toward multi-modal platforms that bypass these gatekeepers entirely, potentially using AI-driven Graph Neural Networks (GNN) to design ligands that exploit receptor-mediated transcytosis more efficiently.

### 5.3. Synaptic Co-Option: Neuronal Activity as a Driver of Malignancy

Recent research has redefined the “neuro-glioma” interface, identifying tumors as functionally integrated components of the brain’s electrochemical network [128]. This synaptic co-option represents an advanced survival strategy where glioma cells utilize endogenous neurotransmission to speed up disease progression. This mechanism is particularly evident in high-grade gliomas, including pediatric diffuse midline gliomas, where malignant cells integrate into functional circuits more aggressively than in adult counterparts [128,129].

A central mechanism in this interaction is the system *x_c_*− cystine/glutamate antiporter, which causes the release of excess glutamate into the peritumoral environment [129,130]. This accumulation induces neuronal damage while simultaneously establishing a feedback loop of hyperexcitability that supports tumor growth [131,132]. By expressing calcium-permeable AMPA receptors, glioma cells respond to neuronal firing through synaptic-like connections [129,133]. These neuronal-to-glioma signals trigger calcium transients that manage cellular proliferation and the development of tumor microtubes (TMs), which serve as the primary anatomical paths for tumor invasion [129,134]. These findings require a conceptual transition in neuropharmacology toward “neuromodulatory” interventions [131]. For instance, the repurposing of the AMPA receptor antagonist perampanel is currently being explored to decouple the tumor from the brain’s functional architecture [130]. However, the clinical translation of such strategies must carefully balance therapeutic efficacy against the potential for neurological side effects arising from systemic AMPA antagonism [131,135].

While the conceptual framework of “neuromodulatory” intervention is compelling, the therapeutic window may represent a primary constraint for clinical implementation. Systemic AMPA receptor antagonism, although potentially efficacious in decoupling malignant cells from neuronal circuits in preclinical models, may pose a significant risk of collateral impairment to physiological synaptic plasticity and neurocognitive function, capabilities, and faculties that may already be at a deficit in patients with high-grade gliomas. From a translational perspective, the clinical viability of such strategies may be contingent upon either the engineering of localized delivery systems to minimize off-target neurological effects or the molecular identification of glioma-specific AMPA receptor auxiliary subunits that may permit selective targeting without compromising the functional integrity of the broader neural network.

### 5.4. Multi-Omics

While the 2021 WHO classification represented a milestone in molecular neuropathology, emerging evidence suggests that static molecular markers alone may not fully capture the dynamic cellular plasticity and microenvironmental complexity of CNS tumors observed through contemporary single-cell and spatial multi-omics analyses.

Tumor heterogeneity refers to the diversity within cancers, broadly divided into intertumoral (between tumors) and intratumoral (within a single tumor) types. Intertumoral heterogeneity describes variations in cancer type, genetics, and behavior between different patients or different lesions in the same patient. Intratumoral heterogeneity involves distinct subpopulations of cancer cells within a single tumor that differ genetically, epigenetically, and phenotypically. These factors drive treatment resistance and metastasis [136].

The TME plays a crucial role in determining response to treatment. This involves a series of interconnected changes in the cellular landscape, spatial organization, and extracellular matrix composition [110].

The defining morphological feature of GBM is its marked heterogeneity, which is reflected in the previously employed term “multiforme”. This heterogeneity has been identified as a key factor contributing to the strong resistance of tumors to therapy. In recent years, the most notable advances have been made in the identification of distinct, prognostically relevant subtypes through gene expression and transcriptomic analyses [137]. Within GBM tissue, many cells with different properties and levels of resistance to therapy are located. As a result, current treatments can eradicate only a subset of tumor cells, while others survive and ultimately drive tumor recurrence [137].

In recent years, multi-omics approaches have emerged as a pivotal strategy for understanding the complex biology of CNS tumors and for identifying novel therapeutic targets in brain cancer neuropharmacology [138,139]. Multi-omics integrates data from multiple molecular layers, including genomics, epigenomics, transcriptomics, proteomics, and metabolomics, to generate a comprehensive systems-level view OF tumor pathophysiology [139]. This integrative scheme enables the identification of regulatory networks and molecular interactions that cannot be fully captured through the analysis of a single omics layer [138]. In the context of malignant brain tumors, particularly GBM, multi-omics analyses have significantly expanded our understanding of the molecular mechanisms underlying tumor initiation, progression, and therapeutic resistance [12,140]. Integrative studies combining genomic and transcriptomic profiling have revealed critical alterations in signaling pathways such as EGFR, PDGFR, and PI3K/AKT/mTOR, which play central roles in tumor growth, invasion, and survival, as reviewed in [140]. The integration of these molecular datasets facilitates the identification of actionable targets and improves the molecular stratification of patients [12]. One of the most promising developments in this field is single-cell multi-omics, which enables the simultaneous analysis of genomic, epigenetic, and transcriptomic changes at the level of individual cells [141]. This technology provides extraordinary insights into intratumoral heterogeneity, a hallmark of aggressive brain tumors and a major contributor to therapeutic failure. By dissecting tumor ecosystems at single-cell resolution, researchers can identify distinct cellular subpopulations, including tumor stem-like cells and therapy-resistant clones, thereby enabling the development of more precise pharmacological interventions. Another rapidly advancing area is spatial multi-omics, which combines high-throughput molecular profiling with spatial information about the cellular organization within tumor tissue. This approach allows researchers to map interactions between tumor cells, immune cells, and stromal components within the TME [142,143]. In GBM, spatially resolved analyses have demonstrated that these cellular interactions play a critical role in shaping the immunosuppressive microenvironment and promoting tumor progression. Understanding these spatially organized molecular networks may facilitate the identification of novel therapeutic targets that disrupt TME interactions [142]. Furthermore, the integration of multi-omics datasets with artificial intelligence and machine learning represents an important emerging direction in neuro-oncology research. Advanced computational models can analyze highly complex datasets generated by modern sequencing technologies, enabling the identification of predictive biomarkers, molecular signatures of therapeutic response, and previously unrecognized regulatory pathways. The integration of multi-omics data with radiomic and clinical datasets may further improve prognostic modeling and guide personalized treatment strategies [138]. Looking ahead, multi-omics approaches are expected to play a central role in the development of precision neuro-oncology, where comprehensive molecular profiling of tumors is integrated with pharmacogenomic information to tailor individualized therapeutic regimens [12]. In addition, combining multi-omics technologies with advanced experimental models—such as patient-derived organoids and brain-on-a-chip systems—may significantly accelerate the discovery and validation of new neuropharmacological targets and therapeutic compounds capable of effectively crossing the blood–brain barrier [144]. Overall, the integration of multi-omics data with computational biology, spatial transcriptomics, and advanced tumor modeling platforms has the potential to transform the identification of precision therapeutic targets and to accelerate the development of innovative neuropharmacological strategies for the treatment of malignant brain tumors [138,143].

Recent advances increasingly support the concept that CNS tumors should not be viewed as single molecular entities but rather as dynamic ecosystems composed of genetically, epigenetically, metabolically, and spatially distinct cellular states [145]. Consequently, integrated multi-omics approaches are now being used to define biologically and clinically relevant molecular subtypes beyond conventional histopathological classification. Combined genomic, transcriptomic, epigenomic, proteomic, metabolomic, and spatial transcriptomic profiling enables the identification of tumor-state programs associated with invasion, immune evasion, stemness, and therapeutic resistance [146]. In GBM, these approaches have revealed proneural, mesenchymal, classical, and hybrid transcriptional states that dynamically evolve under therapeutic pressure and are strongly influenced by the TME. Importantly, single-cell and spatial multi-omics technologies have demonstrated that these molecular programs frequently coexist within the same tumor, highlighting the limitations of bulk-tissue classification schemes [147].

### 5.5. Artificial Intelligence in Brain Tumor Data Analysis

Artificial intelligence (AI) is increasingly transforming the analysis of brain tumor data by enabling the integration of radiological, histopathological, molecular, and clinical information into unified analytical schemes [148]. This development is particularly important in neuro-oncology, where brain tumors exhibit marked spatial and temporal heterogeneity and complex microenvironmental interactions. AI-based approaches allow the extraction of subtle patterns across multimodal datasets, significantly improving tumor detection, classification, and treatment planning [149].

A major contemporary trend is the use of multimodal AI systems that integrateMRI, digital pathology, genomic, and clinical data. These approaches enable more precise tumor characterization and improved prognostic modeling compared to single-modality systems [56,150]. In gliomas, AI models can non-invasively predict key molecular features such as IDH mutation and MGMT promoter methylation, supporting rapid clinical decision-making and reducing reliance on invasive biopsies [150,151].

Radiogenomics has emerged as a particularly important domain, linking imaging phenotypes with genomic and transcriptomic tumor states. Recent studies demonstrate that radiomics combined with transcriptomic data can identify biologically distinct tumor subtypes, including aggressive stem-like cellular programs associated with poor prognosis [152]. Furthermore, AI-driven radiogenomic models have shown robust performance in predicting glioma molecular subtypes and survival outcomes, highlighting their potential role in precision neuro-oncology [152].

Another rapidly developing field is the integration of AI with spatial and molecular profiling. By combining histopathological features with high-dimensional molecular data, AI systems can identify intratumoral heterogeneity and biologically active regions or niches, such as hypoxic or invasive regions, which are critical determinants of therapeutic resistance [134,151]. These advances are particularly relevant for neuropharmacology, as they may guide targeted therapy selection and improve strategies for drug development. Deep learning has also significantly advanced digital neuropathology. AI models applied to whole slide imaging can perform tumor classification, grading, and molecular inference with high accuracy, while recent emphasis on explainable AI improves interpretability and clinical trust [148,153]. In parallel, foundation models and self-supervised learning are emerging as key technologies, enabling robust performance even in data-limited environments typical for rare CNS tumors [154].

Beyond diagnostic classification, AI is increasingly being applied to systems-level modeling of tumor biology and drug discovery. In particular, GNNs have emerged as highly promising tools for modeling complex biological interactions, including gene-regulatory networks, protein–protein interactions, signaling pathways, and TME communication [155]. Unlike conventional machine-learning approaches, GNNs preserve relational information between biological entities, enabling the identification of previously unrecognized molecular dependencies and therapeutic vulnerabilities [156]. In neuro-oncology, GNN-based models are being explored for predicting drug-target interactions, prioritizing candidate biomarkers, identifying resistance-associated signaling networks, and integrating multi-omics datasets into unified computational frameworks [157]. These approaches are particularly valuable in highly heterogeneous tumors such as GBM, where tumor evolution is driven by dynamic interactions between malignant cells, immune populations, vascular niches, and neuronal signaling pathways.

Beyond diagnostic and prognostic applications, AI-driven approaches are increasingly being employed in computational drug discovery and systems pharmacology. Recent machine-learning frameworks integrating graph neural networks, knowledge graphs, and multimodal biomedical datasets have demonstrated strong potential for predicting drug–drug interactions, identifying novel therapeutic combinations, and prioritizing candidate molecular targets. These approaches are particularly relevant in neuro-oncology, where treatment commonly relies on multimodal therapeutic regimens and where tumor heterogeneity frequently promotes adaptive resistance mechanisms. Advanced graph-based AI models can integrate molecular interactions, signaling pathways, pharmacological profiles, and clinical datasets into unified predictive frameworks, thereby supporting the development of precision therapeutic strategies and personalized treatment selection [158].

Despite their potential and advances, AI and machine-learning (ML)approaches still face important challenges, including the risk of overfitting, limited reproducibility across independent datasets, and biases related to data quality and patient selection. Recent meta-analyses indicate that many AI models still lack external validation and exhibit variability across datasets and institutions [159]. Additionally, differences in imaging protocols, limited availability of annotated data, and a deficiency of rare tumor subtypes block clinical translation.

Although convolutional neural networks, transformer-based architectures, graph neural networks, and multimodal foundation models have shown promising retrospective performance in MRI-based tumor segmentation, molecular-status prediction, radiogenomics, and digital pathology classification, their clinical value remains uneven. Most available studies are retrospective, trained on heterogeneous or single-institution datasets, and affected by variability in imaging acquisition, tissue processing, annotation quality, and patient selection. Therefore, current AI models should not be presented as replacing clinicians, but rather as decision-support tools requiring external validation, prospective testing, explainability, and integration with neuropathological and molecular expertise. Future developments will likely focus on integrating multimodal data into unified predictive schemes capable of supporting personalized treatment strategies in precision neuro-oncology [84,154].

The integration of AI-driven network biology with spatial and single-cell multi-omics may ultimately facilitate the development of adaptive precision neuro-oncology strategies, where therapeutic selection is guided not only by static mutations but also by evolving tumor-state transitions and microenvironmental interactions.

### 5.6. Combination Therapeutic Strategies in Brain Cancer

Most brain tumors are gliomas, which originate in glial cells. GBM is highly resistant to conventional radiation and chemotherapy. As already mentioned, GBM is the most common malignant primary brain and other CNS tumor, accounting for approximately half of all malignant primary brain and other CNS tumors, but only a minority of all primary brain and other CNS tumors.GBM shows a median survival of less than two years, and it prevails in adults over 40, mostly in the 75–84 age group [160]. The current standard of care includes maximal safe surgical resection followed by radiation and chemotherapy with TMZ (75 mg/m^2^) and subsequentlyby adjuvant TMZ (150–200 mg/m^2^) for six cycles [161]. Surgical resection represents a cornerstone of GBM treatment, with the use of modern techniques such as preoperative brain mapping, intraoperative ultrasound, intraoperative MRI, and fluorescence imaging providing precise identification of tumor margins and their removal [161,162]. 

**Figure 1:**
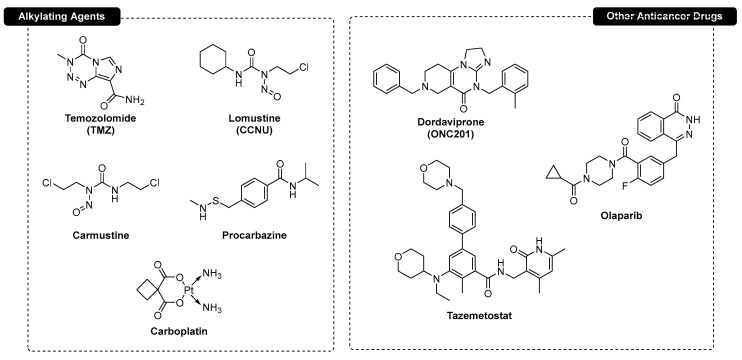
Chemical structures of anticancer drugs employed in clinics.

Among drugs that most successfully pass through the BBB and BBTB, we can list: TMZ, lomustine (CCNU), procarbazine, and carboplatin, whose structures are illustrated in Figure 1 [163].

TMZ, the most widely used lipophilic drug in brain cancer therapy, acts as an alkylating agent, inducing DNA damage through methylation of purine bases. It is mainly responsible for the formation of O6-methylguanine lesions that are responsible for replication errors, apoptosis, autophagy, and cellular senescence of cancer cells. Similar to TMZ, CCNU also exhibits alkylating properties, and its advantage is good penetration of the BBB due to its small size and high lipophilicity. Carmustine is a nitrosourea derivative that induces DNA damage through chloroethylation of guanine residues, ultimately leading to interstrand DNA cross-link formation. It is sometimes administered as a slow-release implant during surgical resection [120,161].

Despite modern therapeutic approaches, the efficacy of brain cancer treatment remains insufficient. This is largely due to various resistance mechanisms, genetic heterogeneity, and the highly invasive potential of cancer cells. Factors contributing to resistance against anticancer drugs include tumor heterogeneity, genetic mutations, epigenetic changes, drug inactivation, efflux of cytotoxic agents, detoxification of alkylating agents, use of alternate signaling pathways, DNA damage repair, impaired ability to commit to apoptosis, modification of drug-binding sites, higher expression of tumor-promoting genes, and various other cellular and molecular mechanisms [71,164]. Consequently, GBM commonly recurs despite multimodal treatment, owing to intrinsic and acquired resistance mechanisms, infiltrative growth, and pronounced intratumoral heterogeneity [163,165]. These treatment limitations highlight the importance of exploring novel therapeutic strategies to enhance the effectiveness of therapies.

A combination of therapeutic strategies has become a central paradigm in the treatment of brain tumors, particularly GBM, where standard monotherapies provide only a limited survival benefit. This is largely due to extensive intratumoral heterogeneity, adaptive resistance mechanisms, and complex TME interactions that collectively limit the efficacy of single-agent therapies [119]. Consequently, rationally designed combination approaches targeting multiple biological processes simultaneously are increasingly being explored. The current standard of care consists of maximal surgical resection followed by radiotherapy combined with TMZ chemotherapy; however, this multimodal approach still results in frequent recurrence and limited long-term survival [166]. Resistance to TMZ is a major challenge and is driven by mechanisms such as enhanced DNA repair and tumor cell plasticity, prompting the development of combination strategies that incorporate additional agents targeting these pathways [167]. Recent preclinical studies demonstrate that combining TMZ with agents affecting DNA replication or repair pathways can produce synergistic antitumor effects and improved survival outcomes [168]. Targeted therapy combinations have also gained significant attention. GBM is characterized by dysregulation of key oncogenic pathways, including EGFR and PI3K/AKT/mTOR signaling, which contribute to tumor growth and resistance [119]. However, single-agent targeted therapies have shown limited clinical success due to pathway redundancy and compensatory signaling. As a result, combination approaches targeting multiple signaling nodes or integrating targeted therapies with radiotherapy are being actively investigated, including biomarker-driven umbrella trials evaluating personalized treatment strategies [169]. Immunotherapy-based combinations represent another major area of development. Although immune checkpoint inhibitors alone have demonstrated limited efficacy in GBM, largely due to the immunosuppressive TME, combination strategies are being explored to enhance immune activation [170]. Approaches combining immunotherapy with radiotherapy, tumor vaccines, or oncolytic viruses aim to improve antitumor immune responses [171]. Radiotherapy, in particular, may enhance immunotherapy efficacy by promoting antigen release and modulating the TME, thereby creating synergistic therapeutic effects [172].

The TME itself has become an important therapeutic target. GBMs exhibit hypoxia, abnormal vasculature, and highly immunosuppressive surroundings, which together limit drug delivery and immune activation [170]. Combination strategies targeting angiogenesis, immune suppression, and stromal interactions are therefore being developed to improve treatment response and overcome resistance mechanisms [173]. Emerging approaches include combinations involving novel modalities such as tumor-treating fields, nanotechnology-based drug delivery systems, and metabolic or epigenetic targeting. Tumor treating fields, when combined with standard therapies, have demonstrated potential to enhance treatment efficacy and are being actively incorporated into multimodal regimens [174]. Similarly, nanomedicine and advanced delivery systems aim to overcome the BBB and improve the effectiveness of combination therapies [167]. Importantly, the success of combination therapies increasingly depends on biomarker-driven patient stratification. Modern clinical trials are moving toward precision oncology schemes, where treatments are tailored based on molecular and genetic tumor profiles, allowing more effective and individualized therapeutic combinations [169]. Without such stratification, combination therapies risk increased toxicity without substantial clinical benefit. Despite these advances, major challenges remain, including treatment-related toxicity, optimal scheduling of combined modalities, and the need for robust clinical validation. Nevertheless, the continued integration of targeted therapy, immunotherapy, and advanced drug delivery systems within biomarker-guided schemes holds significant promise for improving outcomes in brain cancer.

### 5.7. Novel Molecular Targets in Brain Cancer

The identification of novel molecular targets has become a critical focus in brain cancer research, particularly in GBM, where conventional therapies provide limited survival benefits. Advances in molecular profiling have revealed that brain tumors are driven by complex networks of genetic, epigenetic, and metabolic alterations rather than single oncogenic events, demanding the development of targeted therapeutic strategies [12]. These insights have shifted the paradigm from histopathological classification toward molecularly defined tumor subtypes, enabling more precise therapeutic targeting.

One of the most extensively studied molecular targets in GBM is EGFR, which is frequently amplified or mutated, particularly in the EGFRvIII variant. EGFR signaling promotes tumor proliferation, invasion, and resistance to therapy; however, clinical targeting has been challenging due to intratumoral heterogeneity and adaptive resistance mechanisms [175].

Nuclear factor erythroid 2-related factor 2 (NRF2) functions as a master transcription factor orchestrating the expression of an extensive network of genes associated with antioxidant defense and detoxification, thereby serving as a key regulator of cellular redox homeostasis [176]. Beyond its role in redox regulation, Nrf2 acts as a central regulatory node influencing multiple cellular pathways, including cell survival and proliferation, detoxification, metabolism, autophagy, proteostasis, inflammation, immune responses, and cellular differentiation [177]. Many of these pathways depend on the availability of electron-rich metabolic substrates to maintain their functional output. In this context, Nrf2 functions not only as a regulatory orchestrator of these processes but also as a key modulator of glucose and lipid metabolism, aligning metabolic flux with cellular demands [178].

NRF2 protein levels are tightly regulated through Kelch-like ECH-associated protein 1 (KEAP1)-mediated ubiquitination, which targets NRF2 for proteasomal degradation and suppresses its activity under basal, non-stress conditions. Upon oxidative stress exposure, disruption of the KEAP1–NRF2 axis leads to NRF2 stabilization and nuclear accumulation [176], and NRF2 induces the transcription of antioxidant response element (ARE)-driven genes that orchestrate anti-inflammatory responses, redox homeostasis, detoxification pathways, autophagy, and proteasomal function [179]. Through its regulation of antioxidant and detoxification genes, NRF2 protects normal cells against oxidative stress by mitigating the damaging effects of reactive and toxic compounds and is therefore widely regarded as a tumor suppressor [180]. However, in cancer, mutations in KEAP1 [181] and oncogenic drivers such as EGFR, KRAS, BRAF, MYC, and BCR-ABL [182], combined with increased oxidative burden, result in constitutive and aberrant activation of NRF2, promoting tumor adaptation, survival [183], and drug resistance in several cancer types, including brain cancer [184,185,186,187]. Inhibition of NRF2 is anticipated to disrupt redox homeostasis and weaken antioxidant defense mechanisms [188] in brain cancer cells, thereby sensitizing glioma cells to apoptosis. Furthermore, NRF2 targeting may overcome resistance to TMZ, the standard-of-care therapy for glioblastoma [186,189,190,191]. Collectively, these findings position NRF2 as a key therapeutic vulnerability in GBM, as well as in multiple other cancer types.

Another key pathway involves PI3K/AKT/mTOR signaling, which is commonly activated in GBM through genetic alterations such as PTEN loss. This pathway, which also promotes NRF2 activation [192], regulates cell survival, metabolism, and growth, making it an attractive therapeutic target. However, inhibition of this pathway alone has shown limited clinical efficacy, highlighting the need for combination strategies [193]. Moreover, constitutive activation of NRF2 is a common event in cancer that provides a vulnerability that can be targeted through novel drug therapy [194].

A central feature of metabolic reprogramming in cancer is the generation of NADPH, which provides the reducing equivalents required to sustain reductive biosynthesis [195]. Given its central role in sustaining cellular growth and biosynthetic processes, NADPH is produced through multiple pathways operating in both the cytosol and mitochondria. Among these, the pentose phosphate pathway (PPP), IDH1, one-carbon metabolism, and malic enzyme (ME) represent the principal sources of NADPH generation [196]. Relative to normal cells, rapidly proliferating cancer cells enhance the activity of NADPH-producing pathways, allowing them to withstand elevated oxidative stress while meeting the increased biosynthetic demands associated with tumor growth [197]. Among NADPH-producing enzymes, IDH1 [198,199,200], glucose 6-phosphate dehydrogenase (G6PD) [198], and 6-phosphogluconate dehydrogenase (6PGD) [199] have been reported to exhibit upregulation in GBM compared with normal brain tissue [201], suggesting that these pathways play a critical role in sustaining redox balance and metabolic demands in GBM cells.

Modification of heat shock proteins (HSP) is a recognized therapeutic target [202]. Inhibition of HSP70 by antisense has been shown to induce apoptosis in various cancers, including GBM [203]. Activation of glucose-6-phosphate dehydrogenase (G6PD) in GBM is facilitated by HSPB1 via the deacetylase Sirtuin 2 (Sirt2) and subsequently leads to the production of NADPH and pentose, which have been shown to increase proliferation [204]. Methylation-controlled J protein (MCJ) DNAJ heat shock protein family member C15 (DNAJC15) has been implicated in the pathogenesis and resistance to treatment of childhood brain tumors and epithelial ovarian carcinoma [205]. HSP proteins are involved in GBM in promoting angiogenesis (HSP90, HSP47), proliferative activity (HSP60), invasion and initiation of metastasis (HSP90, HSP47), and resistance to apoptosis (HSP90, HSP70, HSP5A, etc.) [206]. ATRX, being a specialized histone chaperone, is involved in genomic instability and alteration in GBM, and its loss in a preclinical study has shown a genetically unstable tumor that is more aggressive but responds better to double-stranded DNA damage, which subsequently leads to increased survival [207]. HSP inhibitors may be effectively applied either as monotherapy or in combination with conventional treatment modalities, such as radiotherapy and chemotherapy [208]. In vivo, HSP90 inhibition by NW457 improved the therapeutic outcome of fractionated irradiation in the GBM mouse model, both in terms of tumor progression and survival [209].

Mutations in IDH1/2 define a distinct subgroup of GBMs and represent one of the most successful examples of molecular targeting in brain tumors. IDH mutations result in the production of the oncometabolite 2-hydroxyglutarate, leading to widespread epigenetic dysregulation. Recently, the IDH inhibitor vorasidenib demonstrated significant clinical benefit in patients with IDH-mutant glioma, marking an important advance in targeted therapy [160].

Approximately 90% of GBMs are classified as IDH-wild, representing the most malignant subtype of glioma [210]. Importantly, nearly two-thirds of high-grade gliomas (HGGs) exhibit significant overexpression of wild-type IDH1 mRNA, defined as greater than a 1.5-fold increase relative to normal brain tissue [211]. Notably, elevated wild-type IDH1 expression shows an inverse correlation with patient survival [212], suggesting that IDH1 may contribute to tumor aggressiveness and represents a clinically relevant metabolic target. Mechanistically, IDH1 targeting disrupts NADPH-dependent redox homeostasis through the depletion of intracellular NADPH pools, thereby limiting glutathione reductase (GR) activity and leading to reduced glutathione (GSH) levels in EGFR-amplified glioblastoma stem cells. These metabolic alterations subsequently enhance cellular sensitivity to EGFR inhibitor-based therapeutic strategies [213].

Radiotherapy, widely applied after surgical resection in combination with chemotherapy, constitutes a standard-of-care approach for glioblastoma [214] but simultaneously induces extensive oxidative stress via ionizing radiation. In response, tumor cells enhance NADPH production to restore redox homeostasis and support the repair of radiation-induced DNA damage, thereby driving the upregulation of NADPH-associated enzymes [215], including IDH1, G6PD, 6PGD, and NADPH oxidase (NOX). The high basal oxidative stress characteristic of glioblastoma, further exacerbated by radiotherapy-induced reactive oxygen species, highlights these enzymes as critical therapeutic targets, either as monotherapies [216,217] or in combination with radiotherapy [201,218,219,220], for cancer treatment.

Epigenetic alterations more broadly have emerged as key contributors to glioma biology. Changes in DNA methylation and histone modification influence gene expression and tumor progression, providing opportunities for therapeutic intervention through epigenetic modulators [221].

Tumor metabolism is another emerging area of interest. GBM cells exhibit metabolic reprogramming that supports rapid proliferation and adaptation to hypoxic conditions. Targeting metabolic pathways, including glycolysis and mitochondrial function, is being explored as a novel therapeutic strategy [222]. In addition, the TME has become an important source of therapeutic targets. GBMs are characterized by an immunosuppressive microenvironment enriched with tumor-associated macrophages and microglia, which contribute to tumor progression and resistance to therapy. Targeting these components, as well as immune checkpoints, represents a promising approach to modulate tumor–immune interactions [223].

Finally, glioma stem-like cells (GSCs) represent a critical therapeutic target due to their role in tumor initiation, resistance, and recurrence. These cells are regulated by developmental signaling pathways, such as Notch, Wnt, and Hedgehog, which are being investigated as potential targets for therapy [224].

Despite these advances, the translation of molecular targets into effective therapeutic strategies remains challenging because of the complex interplay among the multiple biological factors described above. Future progress will likely depend on integrating molecularly targeted approaches with combination treatment strategies and precision medicine frameworks to achieve improved clinical outcomes in patients with brain cancer [225].

Moreover, aldehyde dehydrogenase 1A3 (ALDH1A3) has emerged as a prominent functional marker of GSCs [226] and, more broadly, of CSCs across multiple tumor types, including breast cancer [227]. Briefly, ALDH1A3 belongs to the ALDH1A subfamily, together with 1A1 and 1A2 isoforms, and catalyzes the oxidation of a wide range of endogenous and exogenous aldehydes into their corresponding carboxylic acids. This detoxification activity is essential for protecting cancer cells from reactive aldehydes generated by oxidative stress, lipid peroxidation, and environmental exposure [228,229]. Beyond its cytoprotective role, ALDH1A3 also participates in the final step of retinoic acid (RA) biosynthesis, thereby linking metabolic regulation to differentiation programs and stem cell maintenance [230]. Also, ALDHs play a central role in metabolic reprogramming, with ALDH1A3 being selectively upregulated in the mesenchymal subtype of GSCs, a clinically aggressive form enriched in recurrent tumors and characterized by enhanced glycolytic metabolism, inflammatory signaling, and poor patient outcome. Its expression is tightly associated with stemness features and phenotypic plasticity, supporting the transition toward a therapy-resistant, mesenchymal state [231,232,233]. Functionally, elevated ALDH1A3 levels confer resistance to radiotherapy and TMZ by detoxifying therapy-induced aldehydes and limiting reactive oxygen species (ROS)-mediated cytotoxicity [234]. Moreover, ALDH1A3 has recently been implicated in the regulation of ferroptosis, a form of iron-dependent lipid peroxidation-driven cell death [235].

Beyond its role in therapy resistance, ALDH1A3 actively contributes to tumor progression by promoting angiogenesis, by exerting paracrine effects through the induction of pro-angiogenic factors such as Plasminogen activator inhibitor-1 (PAI-1) and interleukin 8 (IL-8), further reinforcing the aggressive phenotype of mesenchymal GSCs [236]. Thus, the multifaceted role of ALDH1A3 in GBM biology has attracted considerable interest as a therapeutic target.

Importantly, the therapeutic promise of the ALDH1A3 targeting strategy in GBM relies on the selective inhibition of this isoform rather than on broad/pan ALDH inhibition. In fact, as explained, ALDH1A3 functional enrichment in mesenchymal GSCs supports stemness, tumorigenicity, invasiveness, aggressiveness (metastatic potential), and resistance to therapy, including radiotherapy and TMZ. Moreover, normal-tissue expression data from GTEx indicate that ALDH1A3 is detectable in several healthy tissues but is generally low, especially in adult bulk brain samples. Conversely, ALDH1A1 displays a broader expression pattern across normal tissues and brain regions. This distinction highly supports the need to differentiate selective ALDH1A3 inhibition from global ALDH blockade to avoid collateral and toxic side effects. In particular, the availability of 3D structures of both the isoenzymes, with X-ray data providing insights into the catalytic pocket and cofactor-binding sites, enables structure-based drug design (SBDD) approaches and, importantly, the rational development of selective ALDH1A3 inhibitors. Among these, GA11 [237] has demonstrated in vivo efficacy in xenograft models derived from mesenchymal patient-derived GBM cells [238]. Structural data are also available for GA11 and its derivatives in complex with ALDH1A3, revealing that these compounds occupy the apex of the catalytic tunnel, thereby sterically hindering substrate access to the active site [239]. However, ALDH1A3 druggability must be interpreted in light of isoform specificity. ALDH1A3 is not tumor-exclusive and can be detected in several normal tissues, although available bulk-tissue datasets (https://gtexportal.org/) suggest relatively low expression in adult bulk tissues, including brain samples. By contrast, ALDH1A1 displays broader expression across normal tissues and in brain regions. These differences support the rationale for isoform-selective ALDH1A3 inhibition rather than pan ALDH blockade, and this is particularly relevant for safety considerations, given the physiological role of ALDH1A1 in hematopoietic stem/progenitor cells [240].

In this regard, the ALDH1A3-selective inhibitor CLM296 represents an encouraging proof-of-concept, combining low-nanomolar ALDH1A3 inhibition, efficacy in triple-negative breast cancer xenografts, favorable preclinical tolerability, and detectable therapeutic brain exposure after intraperitoneal and oral administration. These findings support its potential relevance for ALDH1A3-positive brain malignancies such as GBM [241].

Thus, despite the intrinsic challenges, including tumor heterogeneity, adaptive resistance, and the restrictive permeability of the BBB, significant progress is being made in translating molecular insights into more effective therapeutic strategies. In this context, the expanding understanding of GBM and other CNS tumors biology, together with the development of highly specific targeted therapies, is driving a shift toward precision medicine, enabling the stratification of patients based on the molecular and phenotypic features of their own tumors and consequently developing a personalized, more tailored, and effective therapeutic intervention. Ultimately, such strategies hold significant promise for improving clinical outcomes in brain cancer by aligning treatments with the specific vulnerabilities of individual tumors [225].

### 5.8. Towards New Medicinal Chemistry Horizons: Multi-Targeting Agents, Theranostics, Probes for Guided-Oncosurgery, and Degraders

Over the past decades, alongside conventional combination therapies, increasing attention has been directed toward the development of single multi-targeting agents. This strategy is particularly relevant in complex diseases such as cancer, where tumor cells rely on multiple dysregulated pathways and progressively acquire resistance mechanisms [242].

Conventional combination therapies, while effective, are often associated with challenges related to dose optimization, pharmacokinetic variability, drug–drug and drug-food interactions, and cumulative toxicity, all of which can complicate clinical management. As a result, there is increasing interest in the rational design of single molecules capable of simultaneously modulating multiple targets involved in disease progression [243].

From a medicinal chemistry perspective, these challenges have driven the development of dual- and multi-targeting agents, commonly referred to as multi-target-directed ligands (MTDLs), which incorporate two or more pharmacophores within a single chemical entity. To achieve this objective, several design strategies have been explored, including the de novo development of small molecules with intrinsic affinity for multiple biological targets, as well as the generation of hybrid compounds through the fusion, merging, or linking of distinct bioactive moieties. In fused hybrids, pharmacophoric elements are structurally integrated into a single scaffold; in linked hybrids, they are connected via a suitable spacer; while in merged hybrids, overlapping structural features are optimized to retain activity toward multiple targets. Multi-targeting strategies also offer a potential solution to the limitations of traditional combination therapies, enabling synergistic or additive therapeutic effects within a single molecular entity [232,244].

Importantly, they may also reduce the emergence of drug resistance by simultaneously interfering with compensatory signaling pathways, representing a significant advantage in highly heterogeneous and adaptive tumors such as GBM.

Several examples have been reported in the literature, with compounds at different stages of development [245,246,247,248].

Beyond target modulation, recent research efforts have increasingly focused on integrating diagnostic capabilities with enhanced therapeutic monitoring, leading to the emergence of theranostic agents. Theranostics refers to the incorporation of both diagnostic and therapeutic functions within a single molecular platform, thereby enabling the simultaneous detection, monitoring, and treatment of disease [249].

This approach can be considered an evolution of conventionally targeted diagnostics. Traditional strategies typically rely on targeting moieties, such as antibodies, peptides, or small molecules, to selectively deliver imaging probes to diseased tissues. In contrast, theranostic platforms incorporate both a diagnostic component and a therapeutically active payload within the same construct. In some cases, the targeting moiety itself may also exert a direct therapeutic effect, beyond its role in mediating selective accumulation.

Theranostic platforms are particularly aligned with precision medicine, as they enable real-time assessment of drug biodistribution, target engagement, and therapeutic response, allowing for integrated feedback. This has important clinical implications, including the potential to optimize dosing regimens, refine patient stratification, and dynamically adapt treatment strategies based on individual responses. In addition, theranostic approaches facilitate in vivo evaluation of drug pharmacokinetics and may guide the subsequent optimization of molecular structure, formulation design, and routes of administration [250]. Representative applications of these strategies include targeted MRI- and positron emission tomography (PET)-based theranostic systems employing nanomaterials [251], as well as contrast or radiolabeled agents selectively delivered to tumor tissues, enabling the simultaneous visualization and treatment of disease [252,253,254].

For instance, multifunctional platforms combining a targeting ligand with a chelating moiety for gallium or gadolinium can serve as PET or MRI contrast agents, respectively, while simultaneously exerting therapeutic effects [255,256]. Notably, gadolinium-containing systems have also been explored in the context of gadolinium neutron capture therapy (Gd-NCT), where the metal ion contributes directly to the therapeutic outcome upon neutron irradiation [257].

Similarly, boron-containing compounds represent a well-established class of theranostic agents in boron neutron capture therapy (BNCT). In this approach, boron-10 isotopes selectively accumulate in tumor cells and, upon neutron irradiation, undergo nuclear reactions that generate high-energy particles, leading to localized cytotoxicity while sparing surrounding healthy tissue [258,259]. Beyond BNCT, increasing attention has been directed toward additional theranostic strategies, including radiolabeled isotope-based systems [260], as well as approaches involving photoactivated chemotherapy (PACT) [261], photodynamic [262], or photothermal [263] therapies, owing to their potential for integrated diagnostic and therapeutic applications [264].Despite their considerable promise, the development of such platforms remains challenging because their design and synthesis often require complex optimization processes, including the determination of appropriate spacer lengths between functional domains and the careful refinement of chemical and metabolic stability, as well as overall pharmacokinetic properties.

In parallel, the development of probes for guided oncosurgery, commonly referred to as fluorescence-guided surgery (FGS), is highly attractive. This approach relies on molecular tools, such as fluorescent probes, to assist surgeons in the intraoperative visualization of tumor tissue. FGS is particularly relevant for highly infiltrative malignancies such as GBM, where tumor margins are poorly defined and difficult to distinguish from surrounding healthy brain tissue. Given that surgical resection remains the first and most critical step in the management of many solid tumors, maximizing the extent of tumor removal is essential for improving patient outcomes [265].

In this context, FGS holds significant promise to increase resection accuracy, reduce residual disease, and ultimately improve patient survival. Notably, it can be considered a clinically relevant application of theranostic principles, where imaging and therapeutic decision-making converge in real time [266].

Clinically used FGS approaches, such as 5-aminolevulinic acid (5-ALA) and fluorescein, have improved intraoperative tumor visualization [267].

However, these agents still present several limitations, including shallow tissue penetration, autofluorescence, and non-specific dye accumulation, which can compromise imaging accuracy and sensitivity. The design of next-generation fluorescent probes for GBM increasingly incorporates targeting moieties, and in some cases, combines targeting and therapeutic functionalities within the same construct.

The selection of an appropriate probe is also critically dependent on its optical characteristics, particularly emission wavelength and tissue-specific properties that influence light propagation, such as scattering by cellular structures and absorption by endogenous chromophores, including hemoglobin and melanin. Indeed, newer FGS probes are designed to operate at longer wavelengths, which enables deeper tissue penetration. In particular, regions within the near-infrared (NIR) spectrum, namely NIR-I (700–900 nm) and NIR-II (1000–1800 nm), are considered “phototherapeutic windows”, where light absorption and scattering are minimized, resulting in improved signal-to-noise ratios and enhanced visualization of deeper tumor regions [268].

Within this framework, ALDH1A3-targeted strategies provide a compelling example of FGS approaches. In particular, curcumin-based probes reported by Gelardi et al. have been designed to selectively target ALDH1A3-expressing cells while enabling fluorescence brightening [269].

However, a notable example of a multifunctional platform is provided by the development of a bimodal imaging platform which integrates a positron-emitting radionuclide for PET with an NIR fluorophore (IR800CW) for FGS, along with an antibody targeting endothelin A receptors (ET_A_), overexpressed in GSCs [270].

Finally, among the latest strategies to tackle GBM, the development of protein degraders is emerging as a particularly promising approach. In particular, proteolysis-targeting chimeras (PROTACs) are based on a targeted protein degradation platform that aims to selectively eliminate proteins involved in disease biology. These systems are heterobifunctional molecules designed to simultaneously bind a protein of interest and an E3 ubiquitin ligase, thereby promoting ubiquitination of the target protein and its subsequent degradation via the proteasome system.

Unlike conventional inhibitors, which are mostly reversible and rely on transient blockade of protein activity, PROTACs enable the complete removal of the target protein, potentially resulting in more effective therapeutic responses. By eradicating oncogenic drivers rather than merely inhibiting them, PROTACs offer the opportunity to overcome resistance mechanisms, target previously undruggable targets, and modulate complex signaling networks [271].

Similarly, molecular glues have recently emerged within the framework of targeted protein degradation in GBM. These agents are typically small monovalent molecules that promote or stabilize protein–protein interactions (PPIs) between a protein of interest and an E3 ubiquitin ligase, thereby inducing their proximity and triggering ubiquitination followed by proteasomal degradation of the target protein [272].

However, despite the rapid clinical development of targeted protein degraders in oncology, PROTAC translation in GBM remains largely preclinical. Several PROTACs have been tested in GBM models, including CDK4/6 and HDAC6-directed approaches, but none have yet reached clinical validation as a GBM therapy [273,274,275,276].

This gap reflects the specific complexity of CNS biology and drug delivery rather than a general failure of modality. Indeed, many PROTACs are large, flexible, and polar molecules, features that may limit BBB and/or blood–tumor barrier (BTB) penetration, oral bioavailability, intracellular exposure, and metabolic stability [277].

In GBM, these limitations are further amplified by the heterogeneous permeability of the BBB/BTB, particularly at infiltrative tumor margins, where drug exposure may be lower than in contrast-enhancing regions [121]. Additional challenges include the need for adequate E3 ligase expression in tumor cells, possible off-target degradation, on-target toxicity in normal brain tissue, hook-effect-related loss of efficacy at high concentrations, and resistance mechanisms affecting the ubiquitin–proteasome system [277].

Importantly, brain exposure is achievable for selected degraders, as shown by the Bruton tyrosine kinase (BTK) degrader NX-5948 in preclinical intracranial lymphoma models [278] and by EGFR degraders such as CFT8919 in non-small-cell lung cancer (NSCLC)brain-metastasis models [277]. Nevertheless, these examples do not directly solve the GBM-specific challenge, where effective drug levels must be achieved across highly infiltrative tumor regions and within a distinct molecular and cellular microenvironment.

Molecular glues represent a related but distinct strategy. Compared with classical PROTACs, they are generally smaller and monovalent, which may offer a more favorable drug-like profile for CNS applications [279]. However, they are less modular and less predictable, as their activity depends on the induction or stabilization of specific protein–protein interactions and may result in degradation or functional perturbation [279].

In GBM, velcrin molecular glues have recently shown promising preclinical activity, leading to cell apoptosis. Notably, one of the developed velcrins was reported to cross the BBB and induce tumor regression in an orthotopic GBM xenograft model. However, this strategy remains biomarker-dependent, requiring adequate PDE3A and SLFN12 expression, and has not yet been clinically validated in GBM [272].

Therefore, while PROTACs and molecular glues are conceptually attractive, their clinical implementation in GBM remains premature. PROTAC translation will require brain-penetrant molecular design and dedicated delivery strategies along with a robust brain pharmacokinetic/pharmacodynamic (PK/PD) validation, whereas molecular glues still require stronger mechanistic characterization and clinical validation.

## 6. Conclusions

CNS tumors remain among the most complex and challenging malignancies because of their marked heterogeneity, aggressive clinical behavior, and limited response to conventional therapies. The shift from purely histopathological classification to integrated molecular diagnostics, as reflected by the 2021 WHO Classification of CNS Tumors,, has substantially improved tumor definition and patient stratification. However, this framework does not fully capture the spatial, temporal, and cellular heterogeneity that characterizes tumors such as GBM, where single-sample diagnosis may underestimate clinically relevant subclones and evolving resistance mechanisms.

In this scenario, multi-omics integration, spatial and longitudinal profiling, liquid biopsy, and AI-based analytical tools may help refine diagnosis, identify actionable vulnerabilities, and support biomarker-driven therapeutic strategies. Nevertheless, these approaches should not be viewed as standalone solutions, as their clinical impact will depend on robust validation, standardization, and integration with histopathological and molecular data. Similarly, emerging therapeutic strategies, including targeted agents, multi-target compounds, theranostics, and protein degradation approaches, remain promising but face major translational barriers, particularly BBB/BTB penetration, tumor heterogeneity, and therapeutic window limitations.

Overall, four take-home messages emerge from this analysis:

- CNS tumor diagnosis is progressively moving from morphology-centered classification (e.g., by WHO 2021) toward integrated approaches incorporating molecular and methylome profiling.
- Tumor heterogeneity and cellular plasticity continue to represent major challenges that limit both diagnostic precision and therapeutic efficacy.
- The evaluation of emerging therapeutic strategies should extend beyond target relevance and also consider critical parameters such as BBB/BTB permeability, brain exposure, safety profiles, and therapeutic-window limitations.
- AI and multi-omics approaches offer powerful tools for tumor stratification and target discovery, but their clinical implementation requires standardization, external validation, prospective testing, and integration with expert neuropathological interpretation.

Future advances in neuro-oncology will therefore require a multidisciplinary and precision medicine-oriented strategy that combines integrated diagnostics, computational modeling, mechanistic validation, and rational therapeutic development. Only through this critical integration will it be possible to move beyond descriptive molecular classification and achieve clinically meaningful improvements in patient outcomes.

## Figures and Tables

**Table 1:** Immunohistochemical markers in CNS tumors, according to the WHO 2021 framework [13].

Tumor Type	Marker	Full Name	Diagnostic Role/Notes
**Glial**	GFAP	Glial Fibrillary Acidic Protein	Principal glial marker; not entirely specific (also in reactive astrocytes), and may show decreased expression with increasing tumor grade, potentially leading to diagnostic challenges in poorly differentiated non-astrocytic mimickers.
	Olig2	Oligodendrocyte Transcription Factor 2	Marker of oligodendroglial lineage, but high sensitivity is occasionally compromised by cross-lineage positivity in certain astrocytic tumors; must be interpreted alongside ATRX status to definitively resolve the lineage threshold.
	S-100	S100 Calcium-Binding Protein	Non-specific; expressed in multiple neural crest-derived cells, exhibits high sensitivity but extremely low specificity in poorly differentiated tumors, frequently necessitating replacement by highly specific molecular panels in clinical practice
	ATRX	α-Thalassemia/Mental Retardation Syndrome X-linked	Loss supports astrocytic lineage (IDH-mutant astrocytoma)
**Neuronal**	Synaptophysin (SYP)	Synaptophysin	Marker of neuronal differentiation, but can show focal positivity in non-neuronal tumors like ependymomas, requiring careful architectural correlation.
	NeuN	Neuronal Nuclei	Mature neuronal marker
	NSE	Neuron-Specific Enolase	Less specific neuronal marker
	NF	Neurofilament Protein	Axonal differentiation
	MAP-2	Microtubule-Associated Protein 2	Dendritic marker
	β-tubulin	β-tubulin	Early neuronal differentiation
**Meningeal**	EMA	Epithelial Membrane Antigen	Commonly expressed in meningioma, typically with membranous staining; perinuclear dot-like or ring-like staining is more characteristic of ependymoma. Essential for differentiating meningioma from schwannoma (EMA-negative), though atypical focal expression in high-grade lesions can mimic metastatic carcinomas.
	Vimentin		Strong but non-specific mesenchymal marker
	PR	Progesterone Receptor	Frequently positive in meningiomas
	SSTR2A	Somatostatin Receptor 2A	Sensitive marker for meningioma
**Choroid plexus**	CK	Cytokeratins	Supports epithelial differentiation
	Transthyretin		Relatively specific marker
	S-100		Variable expression
**Pituitary**	Hormonal markers, e.g., ACTH, GH, PRL		Define functional subtype
	Transcription factors, e.g., PIT1, TPIT, SF1		Lineage specification
	Synaptophysin		Neuroendocrine differentiation
	Chromogranin A		Neuroendocrine marker
	CK	Cytokeratin	Variable expression
**Schwann cell**	S-100		Strong, diffuse positivity
	SOX10	SRY-Box Transcription Factor 10	Sensitive Schwannian marker
**Embryonal tumors**	Variable individual markers		Marker profile depends on subtype
**Germ cell**	SALL4	Spalt-like Transcription Factor 4	Sensitive germ cell marker
	PLAP	Placental Alkaline Phosphatase	Germinoma marker
	AFP	α-fetoprotein	Yolk sac tumor
	HCG	Human Chorionic Gonadotropin	Choriocarcinoma component
	CD30	Cluster of Differentiation 30	Embryonal carcinoma
	CD117	Cluster of Differentiation 117	Germinoma marker
**Melanocytic**	HMB-45	Human Melanoma Black 45	Specific melanocytic marker
	Melan-A		Melanocytic differentiation
	S-100		Highly sensitive but non-specific
	SOX10	SRY-Box Transcription Factor 10	Sensitive melanocytic marker
**Lymphomas**	LCA	Leukocyte Common Antigen	Identifies hematolymphoid origin
	B-cell markers, e.g., CD20, PAX5		B-cell lineage (most CNS lymphomas)
	T-cell markers, e.g., CD3		T-cell lineage
**Vascular**	CD31	Cluster of Differentiation 31	Endothelial marker
	CD34	Cluster of Differentiation 34	Endothelial/vascular marker
**Other mesenchymal**	Variable individual markers		Depending on the histologic origin
**Prognostic markers**	Ki-67	Kiel University, clone 67	Reflects proliferative activity and tumor grade; however, its interpretation may vary across regional hotspot areas, potentially leading to inaccurate prognostic assessment, particularly in small biopsy specimens.
	PHH3	Phosphohistone H3	Mitotic activity marker
	p53	Tumor Protein p53	Associated with genomic instability and poor prognosis, missense mutations often cause strong diffuse positivity, while nonsense mutations result in a complete ‘null’ profile, potentially misleading readers
**Molecular markers**	IDH1/2	Isocitrate Dehydrogenase 1/2	Key classification markers
	ATRX	A-Thalassemia/mental retardation syndrome X-linked	Loss supports astrocytoma
	BRAF	B-Raf Proto-Oncogene	Seen in specific tumor subtypes
	EGFR	Epidermal Growth Factor Receptor	Amplification in GBM

**Table 2:** Representative molecular alterations in selected CNS tumor entities according to the 2021 WHO classification [13].

MAJOR GROUP	Tumor Entity	Key Molecular Alterations
**ADULT-TYPE** **DIFFUSE GLIOMAS**	Astrocytoma, IDH-mutant	IDH1/IDH2 mutation; ATRX loss; TP53 mutation; CDKN2A/B homozygous deletion in grade 4 cases
	Oligodendroglioma, IDH-mutant and 1p/19q-codeleted	IDH1/IDH2 mutation plus whole-arm 1p/19q co-deletion; frequent TERT promoter mutation
	GBM, IDH-wildtype	IDH-wildtype diffuse astrocytic glioma with histological features of GBM and/or molecular features including TERT promoter mutation, EGFR amplification, or combined whole chromosome +7/−10 signature.
**PEDIATRIC-TYPE** **DIFFUSE** **LOW-GRADE** **GLIOMAS**	Diffuse astrocytoma, MYB- or MYBL1-altered	MYB or MYBL1 alterations
	Polymorphous low-grade neuroepithelial tumor of the young	MAPK pathway alterations, commonly BRAF V600E or FGFR alterations
	Diffuse low-grade glioma, MAPK pathway-altered	BRAF, FGFR1, or other MAPK pathway alterations
**PEDIATRIC-TYPE** **DIFFUSE** **HIGH-GRADE** **GLIOMAS**	Diffuse midline glioma, H3K27-altered	H3K27 alteration, including H3-K27M altered mechanisms causing H3K27me3 loss
	Diffuse hemispheric glioma, H3G34-mutant	H3G34R/V mutation
	Diffuse pediatric-type high-grade glioma, H3-wildtype and IDH-wildtype	Entity primarily classified by DNA methylation profiling; frequent RTK/MAPK/PI3K pathway alterations
**CIRCUMSCRIBED ASTROCYTIC GLIOMAS**	Pilocytic astrocytoma	KIAA1549–BRAF fusion or other MAPK pathway alterations
	Pleomorphic xanthoastrocytoma	BRAF V600E; frequent CDKN2A/B deletion
**GLIONEURONAL AND NEURONAL TUMORS**	Dysembryoplastic neuroepithelial tumor	FGFR1 alterations
	Diffuse glioneuronal tumor with oligodendroglioma-like features and nuclear clusters	Subgroups identified by DNA methylation profiling (epigenetic classification)
	Rosette-forming glioneuronal tumor	FGFR1 and/or PIK3CA alterations
	Diffuse leptomeningeal glioneuronal tumor	MAPK pathway alterations, including KIAA1549–BRAF fusion; frequent 1p deletion; methylation profile
	Extraventricular neurocytoma	FGFR alterations
**EPENDYMAL** **TUMORS**	Supratentorial ependymoma, ZFTA fusion-positive	ZFTA fusion, most commonly ZFTA–RELA
	Supratentorial ependymoma, YAP1 fusion-positive	YAP1 fusion
	Posterior fossa ependymoma	Posterior fossa group A or B methylation profiles
	Spinal ependymoma, MYCN-amplified	MYCN amplification
**EMBRYONAL TUMORS**	Medulloblastoma, WNT-activated	WNT pathway activation, commonly associated with a CTNNB1 mutation
	Medulloblastoma, SHH-activated	SHH pathway alterations; TP53 status clinically relevant
	Medulloblastoma, non-WNT/non-SHH	Group 3 and group 4 molecular profiles
	Atypical teratoid/rhabdoid tumor	SMARCB1 or SMARCA4 loss/inactivation
	Embryonal tumor with multilayered rosettes	C19MC alteration; less commonly DICER1-related alterations

Cross-entity biomarkers, such as MGMT promoter methylation, TERT promoter mutation, EGFR amplification, CDKN2A/B homozygous deletion, IDH1/2 mutations, and the +7/−10 chromosome signature, should be interpreted within the appropriate tumor-specific diagnostic context rather than as independent tumor entities.

**Table 3:** Key epigenetic mechanisms in CNS tumors.

Mechanism	Function	Alterations	Clinical Relevance	Therapeutic Strategies
DNA methylation	Gene silencing	hypermethylation, IDH mutations, MGMT status	prognosis, treatment response	DNMT inhibitors, IDH targeting
Histone modifications	Chromatin regulation	H3K27M mutation, PRC2 dysregulation	aggressive tumor phenotypes	EZH2 inhibitors, HDAC inhibitors
miRNA	Post-transcriptional regulation	miRNA dysregulation (e.g., miR-21)	biomarkers, gene regulation	miRNA mimics, antagomiRNAs
Chromatin structure	DNA accessibility, 3D organization	chromatin disorganization	global gene expression control	Epigenetic combination therapies

**Table 4:** Established and emerging diagnostic techniques in GBM, with advantages, limitations, and clinical relevance.

Technique	Type	Target	Advantages	Limitations/Drawbacks	Clinical Role
MRI	Imaging	Tumor morphology, perfusion, diffusion	Non-invasive, high anatomical resolution, widely available	Limited specificity, difficulty distinguishing pseudoprogression from recurrence, weak correlation with molecular features	Initial diagnosis, follow-up
PET	Functional imaging	Metabolic activity	High specificity, can distinguish pseudoprogression from recurrence	High cost, limited availability, not fully standardized	Complementary to MRI
Tissue biopsy	Invasive	Histology and molecular profile	Gold standard, comprehensive molecular characterization	Highly invasive with surgical risks, limited for spatial heterogeneity, not suitable for longitudinal monitoring	Definitive diagnosis
ctDNA	Liquid biopsy	Circulating tumor DNA	High specificity, reflects tumor mutations, enables dynamic monitoring, and is easy to detect	Low concentration in GBM, release limited by the BBB, short half-life	Research
Circulating miRNAs	Liquid biopsy	Regulatory RNAs	Relatively stable, easy to detect	Limited specificity, lack of standardization, not specific for GBM	Research
CTCs	Liquid biopsy	Circulating tumor cells	High specificity, comprehensive analysis of DNA, RNA, and proteins	Rare in blood, difficult isolation, and lack of standardization	Research
Extracellular vesicles (EVs)	Liquid biopsy	RNA, proteins, lipids	Protects cargo, BBB-permeable, reflects tumor biology	Not cancer-specific, lack of standardization of isolation	Promising
Circulating nucleosomes/histone post-translational modifications	Liquid biopsy	Epigenetic modifications	High stability, easy to detect	Low specificity, no defined GBM-specific signature	Early-stage research

## Data Availability

No new data were created or analyzed in this study. Data sharing is not applicable to this article.
